# *Gli3* is a negative regulator of *Tas1r3*-expressing taste cells

**DOI:** 10.1371/journal.pgen.1007058

**Published:** 2018-02-07

**Authors:** Yumei Qin, Sunil K. Sukumaran, Masafumi Jyotaki, Kevin Redding, Peihua Jiang, Robert F. Margolskee

**Affiliations:** 1 Monell Chemical Senses Center, Philadelphia, Pennsylvania, United States of America; 2 School of Food Science and Biotechnology, Zhejiang Gonshang University, Hangzhou, Zhejiang, China; University of Colorado School of Medicine, UNITED STATES

## Abstract

Mouse taste receptor cells survive from 3–24 days, necessitating their regeneration throughout adulthood. In anterior tongue, sonic hedgehog (SHH), released by a subpopulation of basal taste cells, regulates transcription factors *Gli2* and *Gli3* in stem cells to control taste cell regeneration. Using single-cell RNA-Seq we found that *Gli3* is highly expressed in *Tas1r3*-expressing taste receptor cells and *Lgr5+* taste stem cells in posterior tongue. By PCR and immunohistochemistry we found that *Gli3* was expressed in taste buds in all taste fields. Conditional knockout mice lacking *Gli3* in the posterior tongue (*Gli3*^*CKO*^) had larger taste buds containing more taste cells than did control wild-type (*Gli3*^*WT*^) mice. In comparison to wild-type mice, *Gli3*^*CKO*^ mice had more *Lgr5*+ and *Tas1r3*+ cells, but fewer type III cells. Similar changes were observed *ex vivo* in *Gli3*^*CKO*^ taste organoids cultured from *Lgr5+* taste stem cells. Further, the expression of several taste marker and *Gli3* target genes was altered in *Gli3*^*CKO*^ mice and/or organoids. Mirroring these changes, *Gli3*^*CKO*^ mice had increased lick responses to sweet and umami stimuli, decreased lick responses to bitter and sour taste stimuli, and increased glossopharyngeal taste nerve responses to sweet and bitter compounds. Our results indicate that *Gli3* is a suppressor of stem cell proliferation that affects the number and function of mature taste cells, especially *Tas1r3*+ cells, in adult posterior tongue. Our findings shed light on the role of the Shh pathway in adult taste cell regeneration and may help devise strategies for treating taste distortions from chemotherapy and aging.

## Author summary

Adult taste cell regeneration is essential for maintaining peripheral taste cells throughout life. The Shh pathway is an important regulator of taste bud development and regeneration in both embryonic and adult stages. We show that the transcription factor *Gli3*, an important effector of the Shh pathway, is expressed in *Tas1r3-*expressing sweet/umami taste receptor cells and *Lgr5-*expressing taste stem cells. Conditional deletion of the *Gli3* gene led to increased numbers of *Tas1r3-*expressing taste cells and *Lgr5-*expressing stem cells along with altered responses to bitter and sweet tastants. Our findings shed light on adult taste cell regeneration and may lead to new treatments of taste disorders associated with aging and chemotherapy.

## Introduction

In mouse tongue taste buds are found in three types of papillae: anterior fungiform (FF), lateral foliate (FO), and posterior circumvallate (CV). The numerous FF papillae each contain a single taste bud, while the two FO and single CV papillae each contain hundreds of taste buds [1, 2]. Each taste bud contains ~50–100 mature receptor cells classified as type I, type II, or type III cells based on morphology and markers. These cells are further classified into functional subtypes that respond to basic taste qualities of sweet, bitter, umami, sour, and salt [3–5].

Embryonic taste papillae development, especially for anterior tongue, has been well-studied [6–8]. Canonical developmental pathways such as Wnt, sonic hedgehog (Shh), Notch, and fibroblast growth factor (Fgf) pathways drive embryonic taste papillae development [reviewed in: 2, 6, 9]. In adult mice, taste cells survive only 3–24 days, necessitating their regeneration throughout life [10]. However, much less is known about the regulation of adult taste cell regeneration, even though it is essential for maintaining the sense of taste throughout life. Adult taste cell regeneration is affected by aging, radiation treatment and chemotherapy, infection, and autoimmune diseases [11–18].

The role of Shh signaling in regulating taste papillae development and taste cell differentiation at the embryonic and adult stages has been well studied [19–27]. In embryos, SHH is a suppressor of taste placode formation in the FF papillae [17, 28] while it promotes development of taste buds in CV papillae [29]. Yet, SHH overexpression in adult taste epithelium induces numerous ectopic FF taste buds but has no major effect in other taste fields [1, 27]. SHH signals through the membrane-bound receptors PTCH1 and SMO to regulate the bi-functional transcription factors GLI2 and GLI3, the principal effectors of the pathway in adults [30–34]. In the absence of SHH signaling, GLI2 and GLI3 are C-terminally truncated to generate transcriptional repressors that are mostly sequestered in the cytoplasm [30, 33, 34]. SHH signaling prevents the proteolysis of GLI2 and GLI3 and promotes their localization to the nucleus, where they regulate the expression of numerous target genes [35–37].

The role of GLI3 in adult taste cell turnover has not been investigated to date. We generated single-cell RNA-Seq data from multiple taste cell subtypes from mouse CV papillae and found that *Gli3* is highly expressed in green fluorescent protein (GFP) marked cells positive for Lgr5 (Lgr5-GFP marked taste stem cells) and in Tas1r3-GFP marked type II taste cells. These results were confirmed using reverse transcription PCR (RT-PCR), in situ hybridization, and immunohistochemistry. Conditional knockout of *Gli3* (*Gli3*^*CKO*^) in CV and FO papillae increased taste bud size and the numbers of *Tas1r3*- and *Lgr5*-expressing taste cells relative to wild-type animals (*Gli3*^*WT*^). Similar changes were observed in *Gli3*^*CKO*^ taste organoids derived from Lgr5-GFP+ taste stem cells. In posterior tongue *in vivo* and in organoids these alterations were accompanied by changes in the expression of *Tas1r3*, *Trpm5*, *Gnat3*, and multiple *Gli3* target genes. In line with changes in taste cell number and gene expression, *Gli3*^*CKO*^ mice showed increased lick and glossopharyngeal (GL) nerve responses to sweet and umami taste stimuli and decreased lick responses to bitter and sour taste stimuli. Our data indicate that *Gli3* is a negative regulator of differentiation and/or survival of taste stem cells and *Tas1r3*+ type II taste cells that influences taste receptor cell composition and function.

## Results

### *Gli3* is expressed in *Tas1r3*+ and *Lgr5*+ taste cells

**Fig 1:**
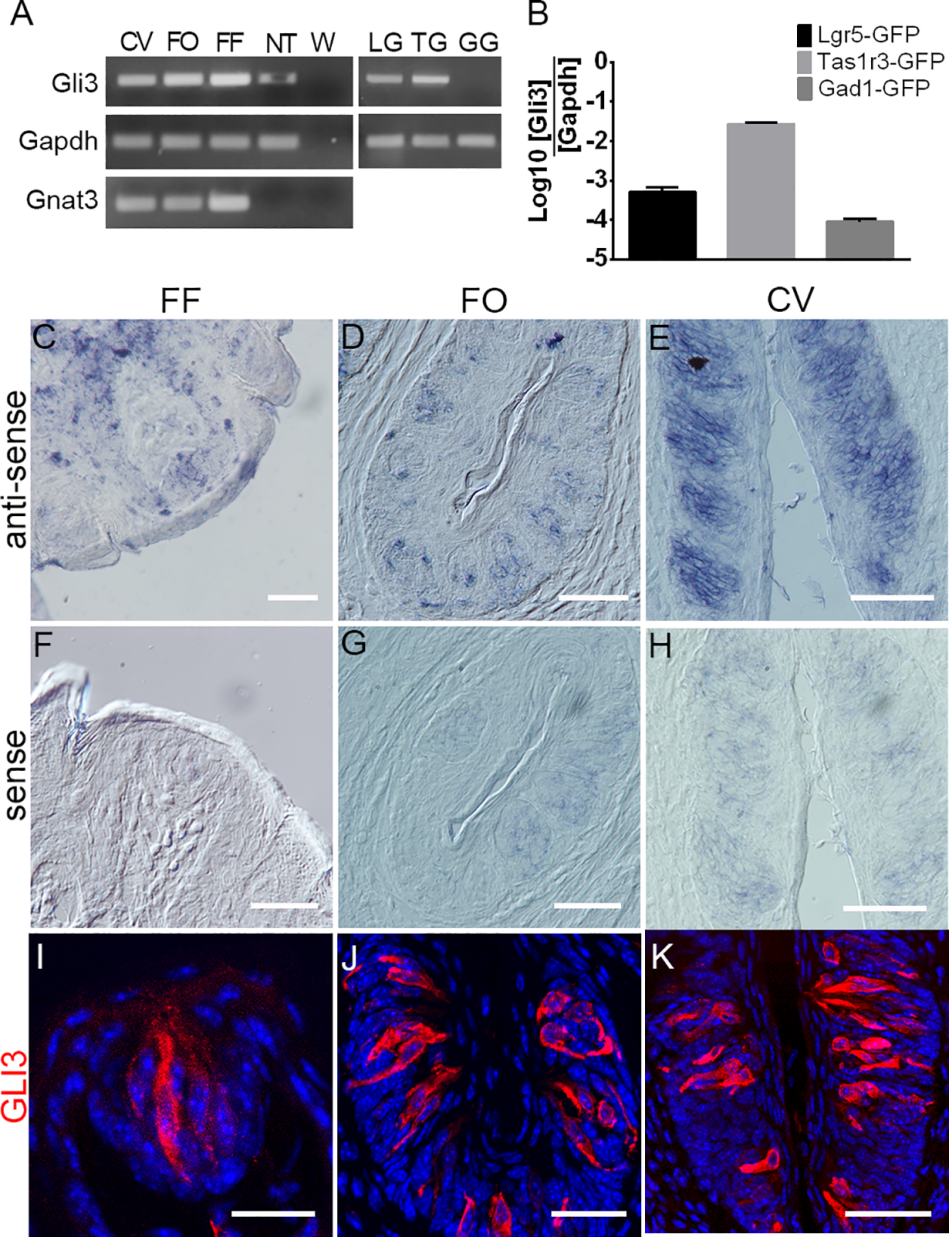
Expression of *Gli3* in mouse taste cells. (A) PCR amplification (35 cycles) of *Gli3*, *Gnat3*, and *Gapdh* from cDNA prepared from circumvallate (CV), foliate (FO), and fungiform (FF) papillae, non-taste lingual epithelium (NT), Lgr5-GFP+ (LG), Tas1r3-GFP+ (TG), and Gad1-GFP+ (GG) cells. *Gli3* is expressed in all taste papillae, NT, Lgr5-GFP+, and Tas1r3-GFP+ cells but not in Gad1-GFP+ cells. (B) qPCR analysis of *Gli3* expression in Lgr5-GFP+, Tas1r3-GFP+, and Gad1-GFP+ cells. *Gli3* is highly expressed in Tas1r3-GFP+ cells, at an intermediate level in Lgr5-GFP+ cells, and at a very low level in Gad1-GFP+ cells. The expression of *Gli3* is plotted as the logarithm of the ratio between its cycle threshold values to those of *Gapdh*. (C-H) *In situ* hybridization using digoxigenin-labeled *Gli3* RNA probes (antisense: C-E; sense controls: F-H) in FF (C, F), FO (D, G), and CV (E, H) showing expression of *Gli3* in taste cells. Signal from sense probe controls in taste cells indicative of nonspecific background was much lower than that of the antisense probe. (I-K) Indirect immunofluorescence confocal microscopy of sections from FF (I), FO (J), and CV (K) stained with a GLI3 antibody shows expression of GLI3 in taste cells. Scale bars: 50 μm.

To identify *Gli*-family transcription factors selectively expressed in subsets of adult taste cells, we analyzed single-cell RNA-Seq data generated from Lgr5-GFP+ stem, Tas1r3-GFP+ type II and Gad1-GFP+ type III taste cells isolated from respective GFP-transgenic mouse strains. The transcription factors *Gli1* and *Gli2* were expressed in all three types of cells, while *Gli3* (and its upstream regulators *Ptch1* and *Smo*) was highly expressed in both Lgr5-GFP+ and Tas1r3-GFP+ but not in Gad1-GFP+ taste cells (S1 Table). RT-PCR showed that *Gli3* was expressed in FF, FO and CV papillae taste tissue, as well as in lingual epithelium devoid of taste buds (Fig 1A). Using GFP+ taste cells purified by fluorescence-activated cell sorting (FACS) *Gli3* was found in Lgr5-GFP+ and Tas1r3-GFP+ but not Gad1-GFP+ taste cells (Fig 1A). Quantification of *Gli3* mRNA expression by quantitative PCR (qPCR) showed that it is expressed at high levels in Lgr5-GFP+ and Tas1r3-GFP+, but at only low levels in Gad1-GFP+ taste cells (Fig 1B). *In situ* hybridization using *Gli3* antisense probes confirmed that it is expressed in taste cells in all three taste papillae (Fig 1C–1E), while the control sense probe produced only minimal background signal in taste cells (Fig 1F–1H). Indirect immunohistochemistry using an antibody generated against the N-terminus of GLI3 capable of detecting both the truncated and full-length forms of the protein revealed that it too is expressed in taste cells in all three taste papillae (Fig 1I–1K). The specificity of RNA probes (S1B and S1C Fig) and antibody (S1E Fig) were validated with the positive control jejunum tissue. Further, both reagents produced weak or no signal in CV papillae from *Skn-1a* knockout mice that lack all type II taste cells (S1D and S1F Fig); pre-incubation of GLI3 antibody with its immunogenic peptide confirmed specificity of the reagent (S1G Fig).

**Fig 2:**
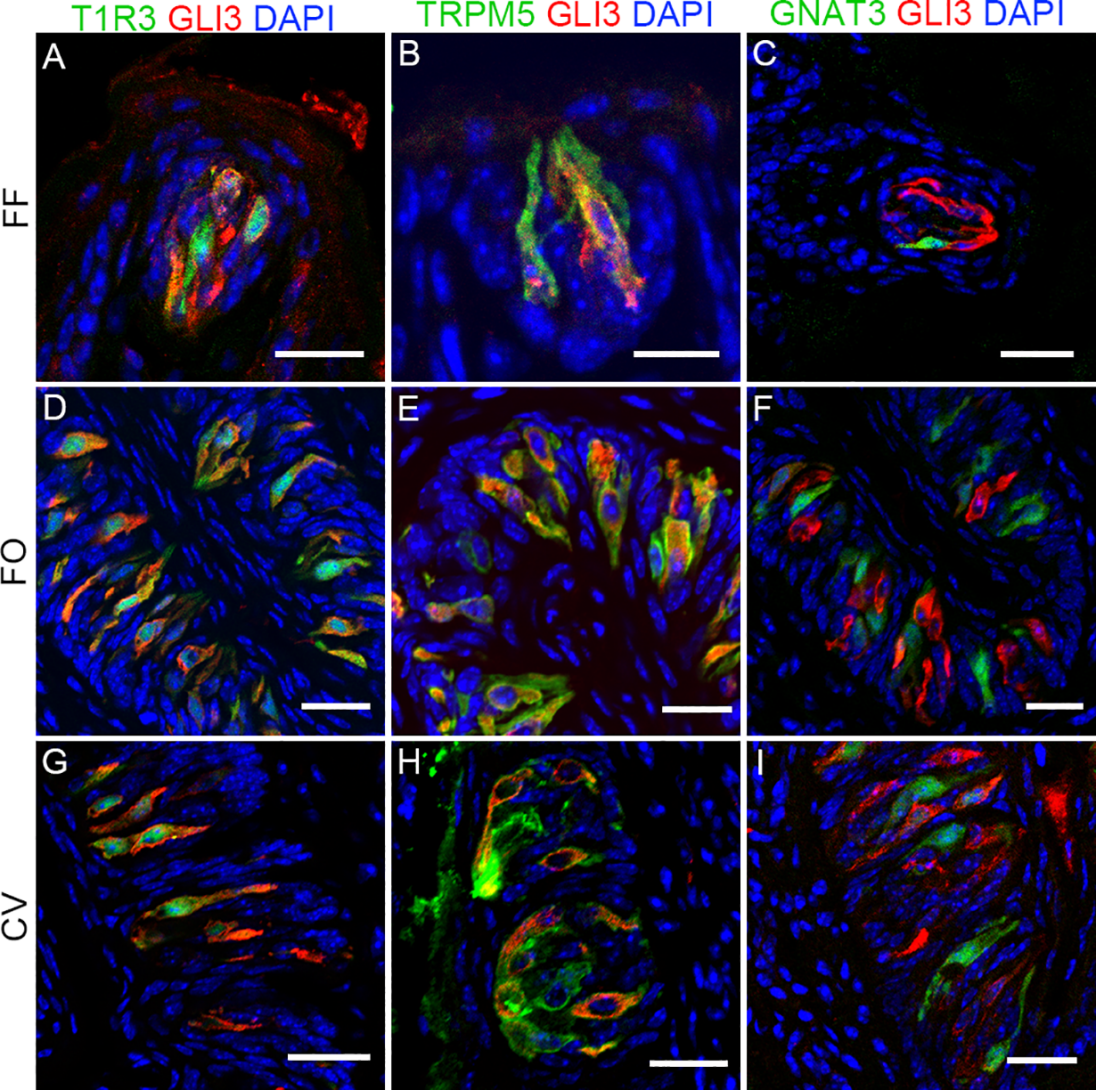
GLI3 is selectively expressed in T1R3+ and TRPM5+ type II taste receptor cells. Double-labeled indirect immunofluorescence confocal microscopy of sections from mouse fungiform papilla (FF; A-C), folate papillae (FO; D-F), and circumvallate (CV; G-I) papillae was performed with antibodies against GLI3 and TRPM5 (B, E, H) or intrinsic GFP fluorescence in *Tas1r3*-GFP (A, D, G) and *Gnat3*-GFP (C, F, I) transgenic mice. Overlaid images show frequent co-expression of GLI3 with *Tas1r3*-GFP and TRPM5 but not with *Gnat3*-GFP. Scale bars, 50 μm.

To confirm these results and identify other taste cell subtypes that express GLI3 we double-labeled taste cells with the GLI3 antibody along with other antibodies or with GFP transgenes that mark specific types of taste cells. In both anterior and posterior tongue fields GLI3 was frequently co-expressed with Tas1r3-GFP, a marker for sweet and umami receptor-expressing type II cells (Fig 2A, 2D and 2G); less frequently with TRPM5, a marker for all type II cells (Fig 2B, 2E and 2H); and at even lower frequency with Gnat3-GFP, a marker for another subset of type II cells (Fig 2C, 2F and 2I). Double-labeled immunohistochemistry with anti-GLI3 antibody plus an antibody against the type III taste cell markers CAR4 (S2A–S2C Fig) and 5-hydroxytryptamine **(**5-HT) (S2D–S2F Fig) or with intrinsic GFP fluorescence of the type I marker Glast1-GFP (S2G–S2I Fig) revealed that GLI3 is generally not expressed in type I or III taste cells.

Quantification showed that GLI3 was frequently expressed with TAS1R3 and TRPM5, but rarely or not at all with CAR4, 5HT or GLAST (S2 Table). Among TAS1R3+ cells, ~92% expressed GLI3 in either CV or FO papillae. With TRPM5+ cells, 45–64% expressed GLI3, with a higher percentage in the CV papillae. For GNAT3+ cells 31–51% expressed GLI3, with a lower percentage in the FO vs. CV papillae taste cells. Only about 3% of CAR4+ and 1% of 5HT+ type III cells also expressed GLI3. While for GLAST+ type I cells, no GLI3+ cells were found among 88 CV and 64 FO papillae taste cells examined. Among the GLI3+ cells, nearly all (97–99%) also expressed TAS1R3 and/or TRPM5, but only 35–63% expressed GNAT3. In sum, GLI3 is expressed in type II cells, most often in the Tas1r3-GFP+ subset and less frequently in TRPM5+ or GNAT3+ subsets.

### Selective ablation of *Gli3* affects taste bud size and composition *in vivo*

**Fig 3:**
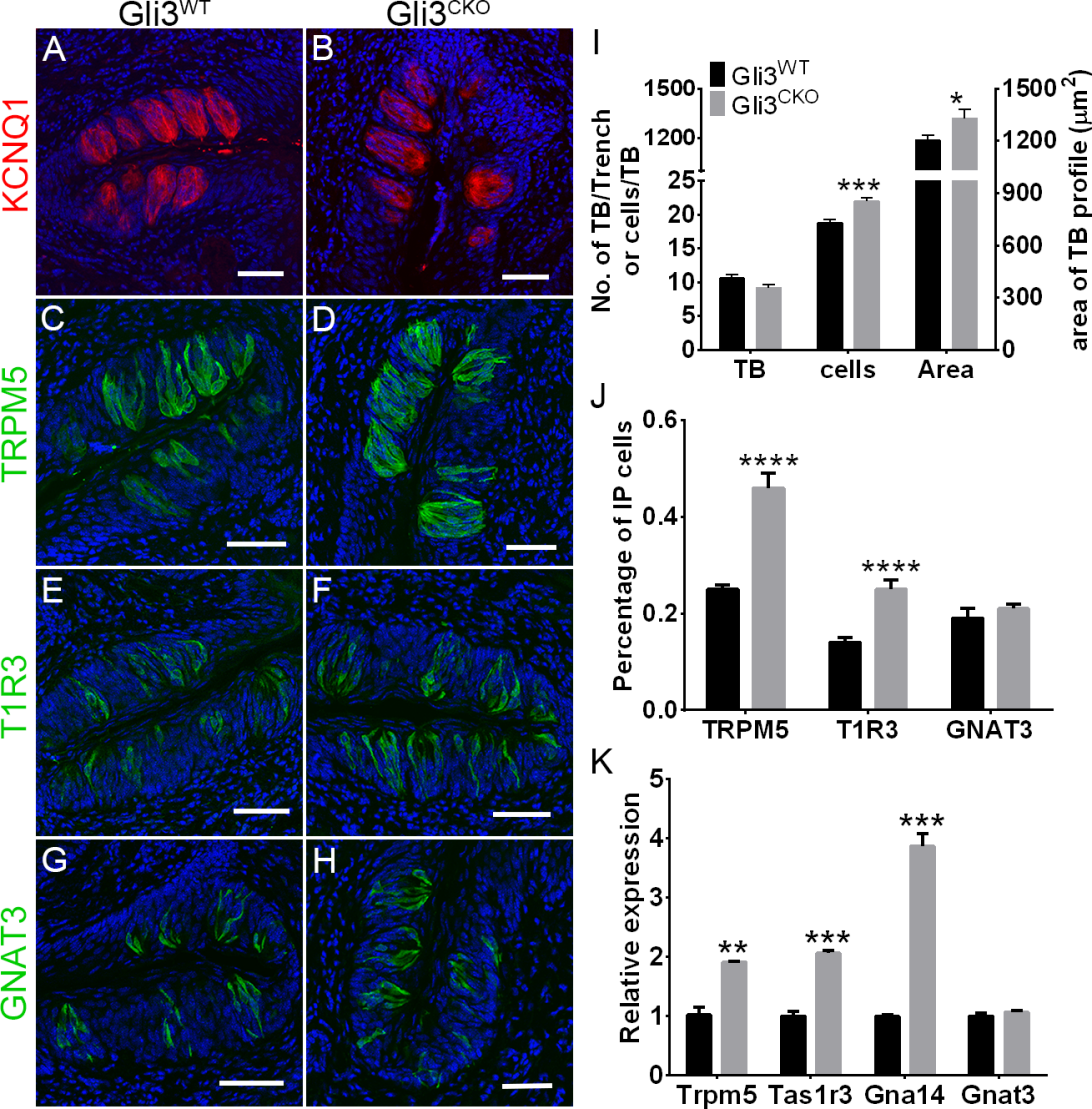
Effect of *Gli3* deficiency on taste bud size and composition. (A-H) Indirect immunofluorescence confocal microscopy of CV sections from (*Gli3*^*WT*^) *Gli3* conditional knockout (*Gli3*^*CKO*^) mice immunostained for all taste cells with antibodies against KCNQ1 (A, B) and immunostained for type II taste cells using markers TRPM5 (C, D), T1R3 (E, F), and GNAT3 (G, H). Nuclei are counterstained with DAPI (blue). Scale bars, 100 μm. (I) Compared to control (*Gli3*^*WT*^) mice, the number of taste buds did not change in *Gli3*^*CKO*^ mice (t = 1.53, p>0.05), but the size (in μm^2^) (t = 2.26, p<0.05) and the number of taste cells (t = 4.58, p<0.001) in taste buds increased. (J) Compared to *Gli3*^*WT*^ mice, the density of TRPM5+ (t = 9.44, p<0.0001) and T1R3+ (t = 8.94, p<0.0001) but not GNAT3+ (t = 1.49, p>0.05) taste receptor cells in *Gli3*^*CKO*^ mice increased. (K) qPCR showed that Trpm5 (t = 6.67, p<0.01), Tas1r3 (t = 12.28, p<0.001) and Gna14 (t = 13.42, p<0.0001) but not Gnat3 (t = 0.87, p>0.05) mRNA expression increased in CV taste buds from *Gli3*^*CKO*^ mice relative to those of *Gli3*^*WT*^ mice. Five control and *Gli3*^*CKO*^ mice each were used for analyses. Data are means + SEM. *p<0.05 **p<0.01, ***p<0.001, ****p<0.0001.

As a key mediator of the Shh pathway that regulates the expression of a large number of genes [35, 37], *Gli3* may play a significant role in taste cell regeneration and survival. To test this, we generated a double-knockin mouse strain homozygous for the floxed *Gli3* allele and also carrying the Lgr5-EGFP-IRES-CreERT2 allele. In this strain, administering the ERT2 ligand tamoxifen would ablate *Gli3* in *Lgr5+* stem cells in posterior tongue. Immunostaining with an antibody for KCNQ1, a marker for all taste cells, showed that in *Gli3* conditional knockout (*Gli3*^*CKO*^) mice, the CV papillae taste buds were larger in size and contained more taste cells than did those of *Gli3*^*WT*^ mice (Fig 3A, 3B and 3I). Most of this may be accounted for by an increase in the number of TAS1R3+ type II cells, as the numbers of TRPM5+ and TAS1R3+ but not GNAT3+ and PKD2L1+ (a type III cell marker) cells increased dramatically in *Gli3*^*CKO*^ mice (Figs 3C–3H, 3J, S3A, S3E and S3I). Conversely, the numbers of CAR4+ type III cells decreased significantly in *Gli3*^*CKO*^ mice (S3B, S3F and S3I Fig). In agreement with this, qPCR showed that mRNAs expressing *Tas1r3*, *Gna14* and *Trpm5*, but not *Gnat3*, *Pkd2l1* or *Snap25*, increased in *Gli3*^*CKO*^ mice (Figs 3K and S3J). As expected, the number of GLI3+ cells and the amount of *Gli3* mRNA decreased drastically, indicating that *Gli3* deletion was successful. At the same time, GFP expression from the *Gli3* locus was turned on in CV papillae tissue from *Gli3*^*CKO*^ mice, which supports this conclusion (S3C, S3D, S3G–S3J Fig). Lgr5-GFP is also expressed in FO papillae (S4A Fig) and changes similar to that in CV papillae were observed in taste buds from FO papillae from *Gli3*^*CKO*^ mice; the number of taste cells per taste bud and the size of taste buds were higher, as were the numbers of TRPM5- and T1R3-expressing type II taste cells (S4B–S4G, S4P & S4Q Fig). Conversely, the number of GNAT3-expressing type II cells and PKD2L1- and CAR4-expressing type III cells did not change; as expected, the number of GLI3-expressing cells were reduced drastically (S4H–S4O, S4P & S4Q Fig). Analysis of FACS-purified cell populations revealed that the proportion of *Lgr5+* taste stem cells increased in *Gli3*^*CKO*^ mice (S5A Fig). Consistent with this, qPCR showed that expression of *Lgr5* mRNA in FACS-purified Lgr5-GFP+ cells (S5B Fig) and in CV papillae (S5C Fig) increased in *Gli3*^*CKO*^ mice. As expected, the level of *Gli3* mRNA in Lgr5-GFP+ cells from *Gli3*^*CKO*^ mice was markedly reduced (S5B Fig). Examining CV papillae taste cells from *Gli3*^*CKO*^ mice showed that the expression of mRNA encoding the *Gli3* target gene *Jag2* decreased, while that for its upstream regulator *Ptch1* increased (S5D Fig). Based on results from selectively ablating *Gli3* from *Lgr5+* cells in posterior tongue, we infer that *Gli3* suppresses the generation or survival of certain subsets of taste cells, in particular the *Lgr5*+ stem and *Tas1r3*+ type II cells in CV and FO papillae, but also the CAR4+ type III cells in CV papillae *in vivo*.

### Selective ablation of *Gli3* affects taste cell abundance *ex vivo*

**Fig 4:**
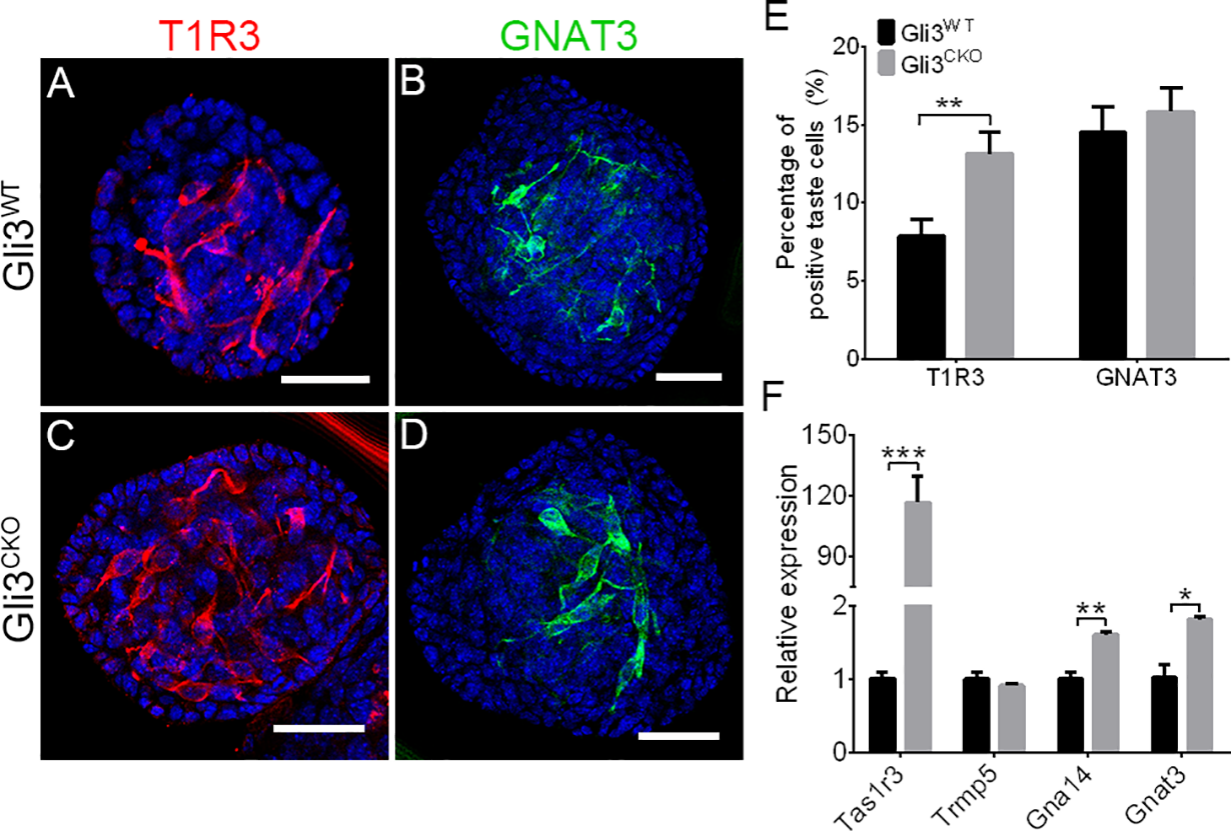
*Gli3* deficiency affects taste cell differentiation and expression of Shh target genes. (A-D) Indirect immunofluorescence confocal microscopy of taste organoids cultured from individual FACS-sorted Lgr5-GFP cells isolated from CV papillae of double-knockin mice treated with tamoxifen (*Gli3*^*CKO*^; C, D) or without (*Gli3*^*WT*^;A, B) and stained with antibodies against T1R3 (A, C) or GNAT3 (B, D). Scale bars, 100 μm. (E) The number of T1R3+ (n = 76, t = 3.05, p<0.01) but not GNAT3+ (n = 84, t = 0.61, p>0.05) cells increased in *Gli3*^*CKO*^ organoids. (F) In *Gli3*^*CKO*^ organoids vs. those from *Gli3*^*WT*^ the expression of *Tas1r3* mRNA (t = 8.87, p<0.001) increased dramatically, while that of *Gna14(t =*, *p<0*.*01)* and *Gnat3* (t = 4.37, p<0.05) showed a modest increase, but that of *Trpm5 (t = 1*.*17*, *p>0*.*05)* remained unchanged. Data are means + SEM. *p<0.05, **p<0.01, ***p<0.001.

Taste organoids cultured from single Lgr5-GFP+ cells faithfully recapitulate many features of taste cell development and function [38]. The effect of *Gli3* ablation on the regenerative potential of Lgr5-GFP+ taste stem cells was tested in taste organoids derived from the double-knockin (floxed *Gli3* Lgr5-EGFP-IRES-CreERT2) mice by adding tamoxifen to the culture medium. Immunostaining of *Gli3*^*CKO*^ organoids showed that the proportion of TAS1R3+ cells increased significantly while that of CAR4+ cells decreased (the proportion of GNAT3+ cells remained unchanged) (Figs 4A–4E, S6A, S6B and S6I). Although not quantified, the proportion of NTPDase-expressing cells appeared largely unchanged (S6C–S6D Fig). Consistent with these results, qPCR of *Gli3*^*CKO*^ organoids showed that mRNAs expressing *Tas1r3*, *Lgr5*, *Gna14* and *Gnat3* increased, while those of *Pkd2l1* and *Snap25* decreased, and that of *Trpm5* and *NTPDase2* remained unchanged (Figs 4F and S6J). Further, the expression of Shh target genes *Gli1*, *Mycn*, *Jag2*, and *Ccnd2* decreased in *Gli3*^*CKO*^ organoids, while that of the upstream regulator *Ptch1* increased dramatically (S6K Fig). As expected, in tamoxifen-treated *Gli3*^*CKO*^ organoids the numbers of GLI3-immunoreactive cells and the level of *Gli3* mRNA decreased, while GFP expression from the *Gli3* locus, indicative of successful *Gli3* deletion, was turned on (S6E–S6J Fig). Collectively, these data suggest that *Gli3* ablation in *Lgr5*+ taste stem/progenitor cells promote expansion or survival of *Tas1r3*+ cells and suppresses the differentiation of CAR4+ type III taste cells.

### Selective ablation of *Gli3* alters behavioral and taste nerve responses

**Fig 5:**
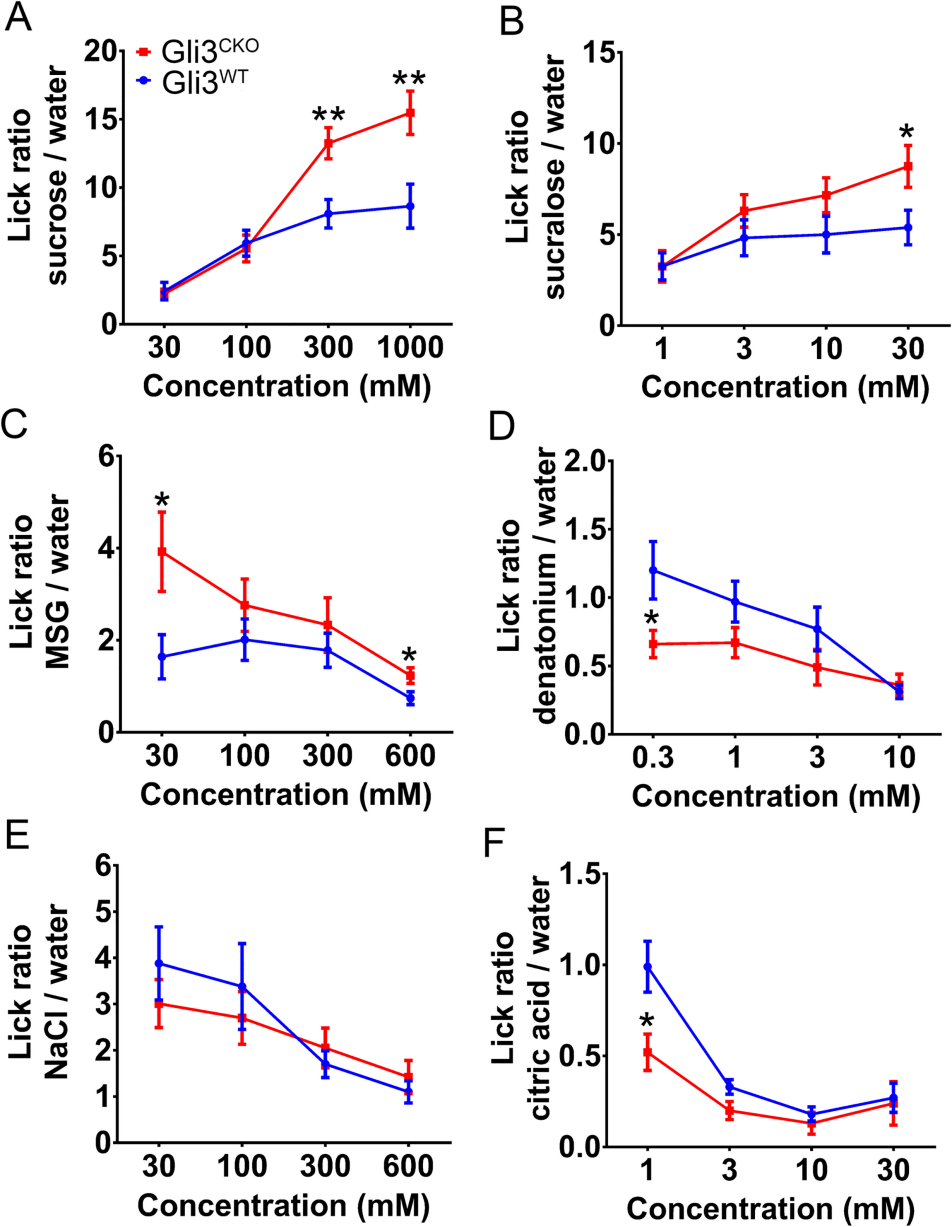
*Gli3*-deficient mice exhibit increased behavioral responses to sweet, umami, and bitter tastants. Brief-access tests were used to measure behavioral responses to sweet (sucrose and sucralose, A and B), umami (monosodium glutamate [MSG], C), bitter (denatonium, D), salty (NaCl, E), and sour (citric acid, F) taste stimuli. Compared to *Gli3*^*WT*^ mice, *Gli3*^*CKO*^ mice display increased lick responses over a range of concentrations to the appetitive stimuli (sucrose, sucralose, and MSG) and decreased lick responses to the aversive stimuli (denatonium and citric acid). Lick ratios were calculated by dividing the number of licks to a taste solution by the number of licks to water in each test session. Data are means ± SEM Statistically significant differences were determined by repeated two-way ANOVA test [sucrose: F (1, 119) = 13.07, P<0.05; sucralose: F (1, 159) = 9.76, P<0.05; MSG: F (1, 119) = 8.69, P<0.05; denatonium: F (1, 123) = 5.40, P<0.05; NaCl: F (1, 147) = 1.57, P>0.05; citric acid: F (1, 127) = 5.45, P<0.05] and post hoc t-test (n>12, *p<0.05, **p<0.01).

**Fig 6:**
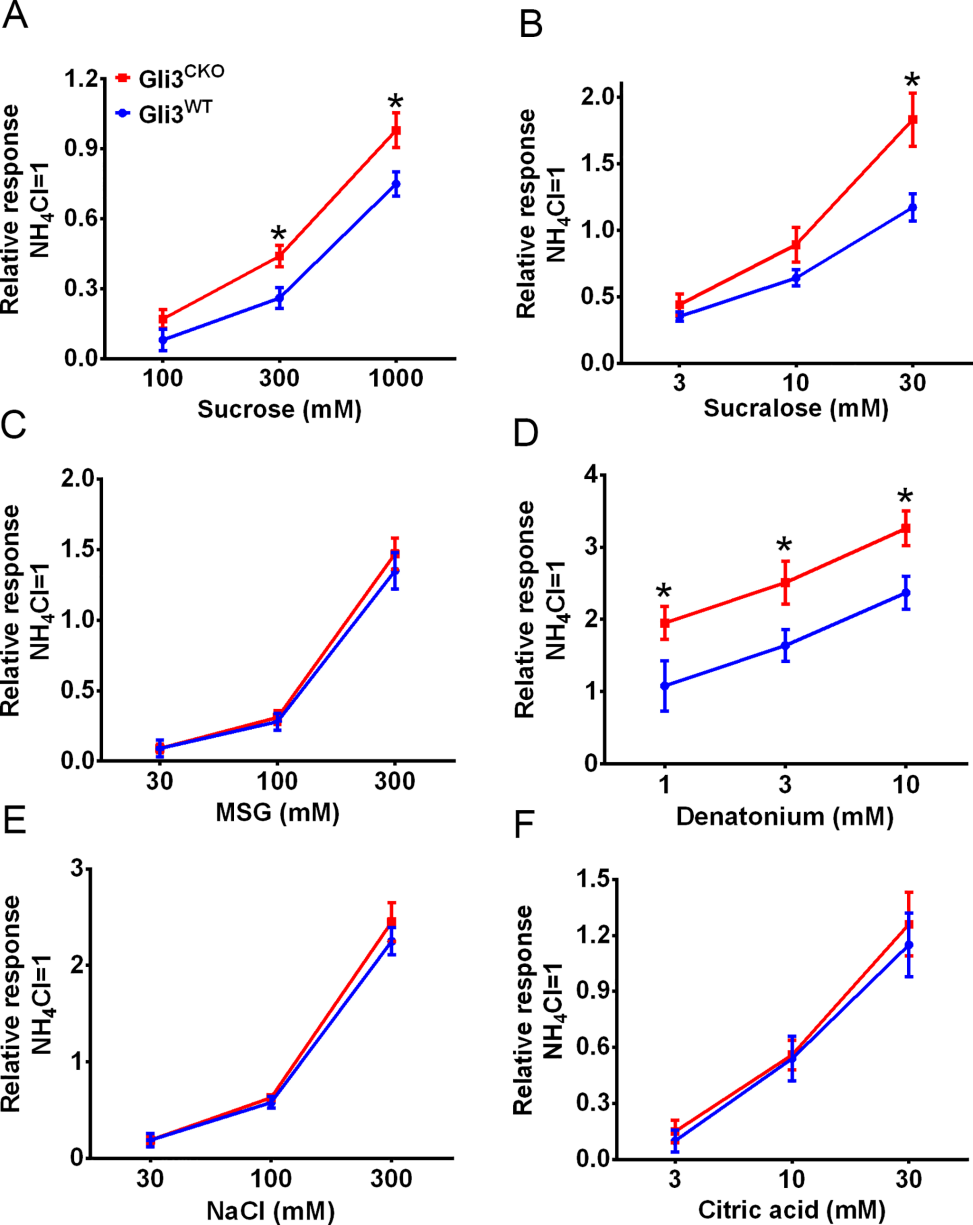
Increased taste nerve responses to sweet and bitter stimuli in *Gli3*^*CKO*^ mice. Average glossopharyngeal (GL) nerve responses to sweet (sucrose and sucralose, A and B), umami (monosodium glutamate [MSG], C), bitter (denatonium, D), salty (NaCl, E), and sour (citric acid, F) tasting compounds are shown. The magnitude of the responses to taste compounds was normalized against the response to 100 mM NH_4_Cl. Compared to *Gli3*^*WT*^ mice, *Gli3*^*CKO*^ mice exhibit increased nerve responses to sucrose, sucralose, and denatonium across a range of concentrations, while the responses to other stimuli were unaffected. Statistically significant differences were determined by repeated two-way ANOVA test [sucrose: F (1, 63) = 12.32, P<0.05; sucralose: F (1, 78) = 9.67, P<0.05; MSG: F (1, 71) = 0.71, P>0.05; denatonium: F (1, 68) = 14.81, P<0.05; NaCl: F (1, 67) = 0.36, P>0.05; citric acid: F(1, 72) = 1.51, P>0.05] and post hoc t-test (n≥7, *p<0.01). All data are presented as the means±SEM.

In light of the profound changes in the proportion of taste cell subtypes and taste gene expression in tamoxifen-treated *Gli3*^*CKO*^ mice, we investigated the effect of *Gli3* ablation on taste responses. In brief-access taste tests, *Gli3*^*CKO*^ mice showed altered behavioral responses to multiple taste qualities. Compared to *Gli3*^*WT*^, the *Gli3*^*CKO*^ mice displayed increased preference for sucrose, sucralose and monosodium glutamate (Fig 5A–5C), increased aversion to denatonium benzoate and citric acid (Fig 5D and 5F), but no change in response to salt (Fig 5E). Glossopharyngeal (GL) nerve recording revealed that compared to *Gli3*^*WT*^ the *Gli3*^*CKO*^ mice had increased nerve responses to sucrose, sucralose, and denatonium (Figs 6A, 6B, 6D and S7). However, the GL nerve responses to monosodium glutamate, citric acid, and NaCl were unchanged in *Gli3*^*CKO*^ vs. *Gli3*^*WT*^ mice (Figs 6E and S7). Furthermore, there were no significant differences in chorda tympani (CT) nerve responses of *Gli3*^*CKO*^ vs. *Gli3*^*WT*^ mice to most of the taste stimuli tested (S8 Fig), consistent with the mosaic expression of *Lgr5-Cre* in the FF papillae taste buds that are innervated by the CT nerve [39].

## Discussion

We used single cell transcriptomics to identify transcription factors selectively expressed in *Tas1r3*+ taste receptor cells, reasoning that they might play a role in the development and/or maintenance of these taste cells. Although the transcription factor *Skn-1a* is critical for the development of *Tas1r3*+ taste receptor cells, it plays this role in all type II taste cells [40]**.** We anticipated that other transcription factors would be expressed selectively in particular subsets of type II cells, e.g. *Tas1r3+* sweet/umami cells or *Tas2r+* bitter cells. By single cell transcriptomics we found that the transcription factor *Gli3* and its upstream regulators *Ptch1* and *Smo* were more highly expressed in *Tas1r3*+ taste cells and *Lgr5+* stem cells than in *Gad1+* type III cells. Conventional expression studies using PCR, in situ hybridization and immunohistochemistry confirmed that *Gli3* was indeed expressed in taste cells. Using *Skn-1a* null mice lacking all type II cells we showed that *Gli3* was expressed selectively in type II cells. By double immunohistochemistry we found that *Gli3* was most highly expressed in *Tas1r3*+ taste cells vs. other types of type II cells (e.g. *Trpm5+* or *Gnat3+* type II cells), and not expressed in type I or III taste cells.

*Gli1*,*2*,*3* are zinc finger-containing transcription factors that act via the Shh pathway to regulate organogenesis and self-renewal [41, 42]. *Gli2* and *Gli3* are the main effectors of the Shh pathway in adults, with *Gli2* acting mainly as a transcriptional activator and *Gli3* as a repressor [33, 34]. Overexpression of *Gli2* leads to malformation of taste buds in FF papillae, while overexpression of a dominant negative *Gli2* transgene or deletion of *Gli2* in taste cell precursors results in loss of taste buds in both FF and CV papillae [19, 43]. However, prior to our work the effects of manipulating *Gli3* on adult taste cell regeneration were not known. To determine what role *Gli3* might play in taste cells we turned to knockout mice. Conventional *Gli3* null mice are embryonic/perinatal lethal [44, 45]; therefore we generated conditional null mice in which *Gli3* was selectively eliminated from taste stem cells using a transgene in which CRE-ERT2 was driven from the *Lgr5* promoter. Conditional ablation of *Gli3* from taste stem cells and their progeny in the posterior taste field led to altered taste bud morphology with numbers of *Tas1r3+* taste cells, but not of *Gnat3+* cells. These changes may be cell-autonomous and cause only an increase in taste bud size at the expense of the epithelial tissue within the taste papillae or cause an overall increase in the size of the taste papillae by affecting the fate of the neighboring non-taste epithelium by non-cell-autonomous mechanisms. We have not tested which of these two possibilities account for the changes in *Gli3*^*CKO*^ mice. The *Gli3*^*CKO*^ mice showed altered short-term lick test responses to sweet, umami, and bitter tastants and diminished glossopharyngeal nerve responses to sweet and bitter. Although Lgr5-CRE-ERT2 is only expressed in a weak, mosaic pattern in FF papillae, we observed modest changes in CT nerve responses to sucralose and citric acid, indicating that Gli3 could play a role in the anterior taste field also. Definitively determining this will require experiments using a Cre driver that is strongly expressed in FF papillae.

The Shh pathway is active in all taste papillae [19, 24, 46, 47], but its effect is context dependent. In the embryonic stage, Shh signaling suppresses the development of FF papillae, while it promotes taste bud development in CV papillae [20, 28, 29, 48]. In adults, Shh expressing cells give rise to all subtypes of taste cells; pharmacological inhibition of Shh signaling inhibits taste cell turnover [21, 24, 47, 49]. Further, overexpression of Shh in the lingual epithelium triggers the development of multiple ectopic FF taste buds [27]. SHH is secreted by a subpopulation of post-mitotic cells in the base of the taste buds, and SHH-responsive, putative stem cells are located around and outside the base of taste buds. Indeed, current evidence suggests that the Shh pathway is active in stem cells [19, 23]; and is critical for the development of taste cells in all taste fields, as noted above. *Lgr5* is a marker for posterior taste stem cells, but also a co-receptor in the Wnt signaling pathway [38, 39]. Because we ablated *Gli3* in posterior tongue using the Lgr5-CreERT2 driver and because the Shh pathway is downstream of Wnt [28, 50] it is likely that we ablated *Gli3* in taste stem cells before Shh signaling was turned on. Using the Lgr5-CreERT2 driver and tamoxifen, *Gli3* was ablated from most but not all posterior field taste cells. The remaining *Gli3+* cells may be progeny of the *Lgr5*+ cells where *Gli3* deletion failed or long-lived taste cells generated prior to tamoxifen treatment.

In *Gli3*^*CKO*^ mice the numbers of type III cells did not change overall, but the *Car4+* subset of type III cells decreased markedly. Notably, *Gli3* is not expressed in type III cells, including *Car4*+ cells, so its effect on this cell type is most likely a consequence of *Gli3* activity in the *Lgr5+* stem cells themselves or in lineage-specific precursor cells that gave rise to *Car4+* cells. It is possible that the lack of *Gli3* inhibits the differentiation of *Car4+* cells as it does not affect the expression of *Car4*.

CAR4 is thought to be necessary for amiloride-insensitive salt taste perception [51], but *Gli3*^*CKO*^ mice retained normal salt taste sensitivity. Conversely, *Gli3*^*CKO*^ mice had heightened sensitivity to all other primary taste qualities in brief-access tests and to bitter and sweet tastants in taste nerve responses. The magnitude of the changes in lick responses in particular are somewhat surprising because the anterior taste field is not affected in *Gli3*^*CKO*^ mice, and may mask the effect of changes in the posterior taste field. But it is possible that taste buds in the soft palate, which also are endoderm-derived and in the pharynx could show changes similar to those in CV and FO papillae in *Gli3*^*CKO*^ mice, although we have not tested this. Consistent with these observations, only those taste qualities that elicit stronger responses in the posterior taste field, namely sweet and bitter, show robust changes in *Gli3*^*CKO*^ mice. On the other hand, the behavioral and GL nerve responses to umami tastants did not change dramatically, although *Gli3*^*CKO*^ mice had a higher number of *Tas1r3+* cells and expressed more *Tas1r3* mRNA than did wild-type mice in CV papillae. This may reflect the low baseline umami taste sensitivity and expression level of the *Tas1r1* subunit of the umami taste receptor in CV papillae [52, 53]. In *Gli3*^*CKO*^ mice the number of bitter (*Gnat3*+) and sour (*Pkd2l1*+) receptor cells did not change, but the sensitivity to these tastants, especially to bitter, increased. This may be attributed at least in part to changes in innervation density or selective innervation of particular types of taste cell types, although we have not tested this. Another possibility is that the expression level of taste receptors or their downstream signaling/regulatory machinery changed in *Gli3*^*CKO*^ mice. Indeed, the expression of many taste marker genes, such as *Tas1r3*, *Trpm5*, *Gnat3*, *Gna14*, *Snap25*, *Pkd2l1* and *NTPDase2*, is affected in *Gli3*^*CKO*^ mice and/or organoids.

In CV papillae and/or organoids derived from *Gli3*^*CKO*^ mice, we observed changes in mRNA expression of the *Gli3* target genes *Ccnd2*, *Mycn*, and *Jag2* and of the upstream regulator *Ptch1*. While these changes confirm that *Gli3* deletion had the expected effects, they represent only a small subset of the thousands of *Gli3* target genes. RNA-Seq analysis of *Gli3*^CKO^ taste cells may help identify many more genes affected by *Gli3* deletion and help delineate the developmental pathways regulated by *Gli3* in taste cells.

In this study we demonstrate the utility of organoids cultured from purified taste stem cells for studying taste system development. Being an *ex vivo* system, taste organoids are not influenced by signals from other tissues. Hence, the results of genetic or other manipulations can be interpreted in a more straightforward manner. Also, the role in the taste system of key genes and pathways can be readily studied in taste organoids without concern for lethal effects from knockouts *in vivo*. Further, large numbers of cultured taste cells can be obtained from organoids which will be useful for protein expression and biochemical studies. Indeed, the effect of *Gli3* knockout in taste organoids largely parallels that observed *in vivo*, underlining the utility of this system.

What could be responsible for the increases in *Lgr5+* and *Tas1r3+* cells in *Gli3*^CKO^ mice? In other tissues, Shh signaling can drive either differentiation or maintenance of stem cells [54, 55]. It is possible that *Gli3* enhances taste stem cell maintenance and acts as a negative regulator of taste cell differentiation. Another possibility is that *Gli3* promotes apoptosis of *Tas1r3+* and/or *Lgr5+* cells. In either case, our data support a critical role of *Gli3* activity in both stem and type II sweet taste receptor cells. The continued expression of *Gli3* in *Tas1r3+* cells and the profound changes in the number of these cells and in sweet and bitter taste sensitivity in *Gli3* conditional knockout mice are evidence for an additional role for Shh signaling and *Gli3* in these mature taste cells. One way to tease apart the role of Shh signaling in stem and *Tas1r3+* cells is by conditional ablation of *Gli3* or other Shh pathway components using *Tas1r3*- or type II-specific Cre drivers (e.g. *Skn-1a* [40])**.**

The role of other signaling pathways in taste development can also be context dependent. The Wnt and Bmp signaling pathways are critical for the development of embryonic FF papillae, but play relatively minor roles in the CV papillae [17, 28, 56–58], while the Fgf signaling pathway, much like the Shh pathway, plays opposite roles in embryonic CV and FF papillae development [2]. Such differences are not surprising given that developmentally the FF papillae originate from the ectoderm while the CV and FO papillae are derived from the endoderm [59]. Many of these pathways may play relatively subtle but significant roles in taste fields where they seem dispensable (similar to what we have shown for *Gli3*, and by extension the Shh pathway).

In summary, our results indicate that *Gli3* is a suppressor of taste stem cell proliferation and affects the number and function of mature taste cells, especially of the *Tas1r3*+ subtype in posterior tongue. Our findings shed more light on adult taste cell regeneration and may help devise strategies for treating taste distortions caused by conditions such as chemotherapy and aging.

## Materials and methods

### Animals

All animal experiments were performed in accordance with the National Institutes of Health guidelines for the care and use of animals in research and approved by the Institutional Animal Care and Use Committee at Monell Chemical Senses Center (protocols: #1163, #1151). 6-12-week old were used for all experiments. Animals were housed with a 12-h light/dark cycle and *ad libitum* access to food and water. The double-knockin mouse strain carrying a floxed *Gli3* allele was a kind gift from Dr. Rolf Zeller, University of Basel (Basel, Switzerland) [60]. Lgr5-EGFP-IRES-CreERT2 knockin mice and *Tas1r3-*GFP and *Gnat3*-GFP transgenic mice were as previously described [61, 62]. *Glast1*-EMTB-GFP was a kind gift from Dr. Eva Anton, University of North Carolina School of Medicine (Chapel Hill, NC)[63]. *Skn-1a* knockout mice were a kind gift from Dr. Ichiro Matsumoto, Monell Chemical Senses Center (Philadelphia, PA) [40]. For Cre activation, tamoxifen (Sigma-Aldrich, St. Louis, MO; cat. no. T-5648) was dissolved in corn oil (Sigma-Aldrich cat. no. C8267) to a stock concentration of 20 mg/ml and administrated by oral gavage for three weeks at a dose of 2 mg/20 g body weight. Mice were given 2-day breaks each week during treatment to recover from the drug. Tissue was harvested 4 weeks after completion of tamoxifen treatment.

### Isolation of lingual epithelium

Mice were sacrificed by CO_2_ asphyxiation, and the tongues excised. An enzyme mixture (0.5 ml) consisting of dispase II (2 mg/ml; Roche, Mannheim, Germany; cat. no. 04942078001) and collagenase A (1 mg/ml; Roche cat. no. 10103578001) in Ca^2+^-free Tyrode’s solution (145 mM NaCl, 5 mM KCl, 10 mM HEPES, 5 mM NaHCO_3_, 10 mM pyruvate, 10 mM glucose) was injected under the lingual epithelium, which was then incubated for 15 min at 37°C. Lingual epithelia were peeled gently from the underlying muscle tissue and used for single-cell RNA-Seq, FACS sorting, or RNA isolation.

### Single-cell RNA-Seq analysis

Single cell RNA-Seq was done as described [64]. GFP-expressing cells that were not physically attached to any other cell or cell fragment were picked irrespective of their shape individually from single cell preparations of CV papillae of *Tas1r3*-GFP (type II, sweet and umami receptor cells, n = 9), *Lgr5*-GFP (stem cells, n = 5), and *Gad1*-GFP type III, sour and high salt receptor cells, n = 11) transgenic mice. Two rounds of single-cell mRNA amplification were done using the TargetAmp 2-Round aRNA Amplification Kit 2.0 (Epicentre, Madison, WI). The antisense RNA generated from single cells was converted to Illumina sequencing libraries using the NEBNext Ultra Directional RNA Library Prep Kit for Illumina (New England Biolabs, Ipswitch, MA) and sequenced using the Illumina HiSeq 2000 platform. Sequencing reads were mapped to the mouse genome (version mm10, p4) using the STAR aligner [65] using Gencode M7 as splice junction database (https://www.gencodegenes.org/mouse_releases/7.html). The reads mapping to genes were counted using the featureCounts package [66] with Gencode M7 as reference. Data normalization and differential expression analysis were done using the DESeq2 package in R [67]. We obtained 30–70 million reads per library, of which 70–90% could be aligned to the mouse genome. On average, 10,184 genes were expressed per cell above an arbitrary cutoff of 10 reads per gene after normalization.

### Fluorescence-activated cell sorting

GFP-fluorescent *Tas1r3+*, *Gad1+*, and *Lgr5+* taste cells were isolated by FACS from male mice of these respective genotypes. The region of the lingual epithelium containing the CV papillae from four to five mice was excised and pooled, minced into small pieces, incubated with trypsin (0.25% in PBS) for 10–25 min at 37°C, and mechanically dissociated into single cells using heat-pulled Pasteur pipettes. Cell suspensions were filtered using 70-μm cell strainers (BD Biosciences, Bedford, MA; cat. no. 352350) and then with 35-μm cell strainers (BD Biosciences cat. no. 352235). Cells were sorted into culture medium for organoid culture or Trizol LS (Thermo Fisher cat. no. 10296010) for RNA isolation using a BD FACS Aria II SORP FACS machine (Flow Cytometry and Cells Sorting Resource Laboratory, University of Pennsylvania), according to the enhanced green fluorescent GFP (EGFP) or GFP signal (excitation, 488 nm; emission, 530 nm).

### PCR and qPCR

Total RNA was isolated from freshly dissected taste papillae, nontaste control epithelium from the ventral surface of the tongue, and taste organoids using the PureLink mini kit with on-column DNA digestion using PureLink DNase (Thermo Fisher cat. no. 12185010) and converted into cDNA using Super Script VILO kit (Thermo Fisher cat. no. 11755050). RNA from FACS-sorted cells was isolated using the Trizol LS kit, and cDNA was synthesized using Ovation qPCR System (NuGEN, San Carlos, CA; cat. no. 2210–24). End-point PCR and qPCR were done as described [68]. Initially the expression of *Gli3* was plotted as the logarithm of the ratio between its cycle threshold value and that of *Gapdh*. Subsequently, all qPCR results were normalized using the ΔΔCt method with *Bact* as reference.

### 3D taste organoid culture

Taste organoids were prepared as described [38]. Briefly, GFP fluorescent cells sorted from double-knockin mice were mixed with 4% chilled Matrigel (v/v; BD Biosciences, San Jose, CA; cat. no. 354234) and cultured in DMEM/F12 (Thermo Fisher cat. no. 11320–033) supplemented with Wnt3a-conditioned medium (50%, v/v), R-spondin-conditioned medium (20%, v/v), Noggin-conditioned medium (10%, v/v), N2 (1%, v/v; Thermo Fisher cat. no. 17502–048), B27 (2%, v/v; Thermo Fisher cat. no. 12587–010), Y27632 (10 μM; Sigma-Aldrich cat. no. Y0503), and epidermal growth factor (50 ng/mL; Thermo Fisher). Wnt3a- and R-spondin-conditioned medium are generated from Wnt3a and R-spondin stable cell lines as described [69]. Noggin conditioned medium was made in house. The culture medium was changed first at day 5–7 and once every 2–3 days thereafter. For passage, single-cell preparations were made from taste organoids by digestion with 0.25% trypsin for 10 min at 37°Cat day 14 before seeding again onto culture plates. 4-Hydroxytamoxifen (10 μg/ml; Sigma-Aldrich cat. no. H7904) was added into the fresh sorted cells for 5 consecutive days for Cre activation.

### Tissue preparation

Adult male mice were euthanized by CO_2_ asphyxiation, and taste-papillae-containing portions of the tongue were quickly removed and briefly rinsed in ice-cold PBS. For *in situ* hybridization, tissues were freshly frozen in Tissue-Tek O.C.T. mounting media (Sakura Finetek, Torrance, CA; cat no. 4583) using a 100% ethanol dry ice bath and sectioned within 1 h after dissection. For immunohistochemistry, tissues were fixed for 1 h at 4°C in 4% paraformaldehyde in 1× PBS and cryoprotected in 20% sucrose in 1× PBS overnight at 4°C before embedding in O.C.T. Sections (10 μm thickness, coronal for FF and CV papillae, horizontal for FOL papillae) were prepared using a CM3050S cryostat (Leica Microsystems) and applied on precoated Fisherbrand Superfrost microscope slides (Fisher Scientific, Hampton, NH, Cat no 12-550-123). Sections were dried at 40°C for 20 min and immediately used for *in situ* hybridization or stored at −80°C for immunostaining.

### *In situ* hybridization

Standard *in situ* hybridization methods were used as described. Fresh tissue sections with taste papillae were incubated with hybridization of 0.3 μg/ml Gli3 probe (GenBank NM_00813, 1809–2427 bp). Antisense and sense RNA probes were used at equivalent concentrations and run in parallel in the same experiment to ensure equivalent conditions. For each experiment, a positive control hybridization using *Tas1r3* probe was done. In addition, *in situ* hybridization experiments were done on positive control tissues to confirm the quality and specificity of the RNA probes.

### Immunostaining

Immunostaining of taste buds was done as described. The antibodies used in this study and their concentrations are listed in S4 Table. For serotonin detection, mice were injected with 5-HT (Sigma-Aldrich cat. no. H9523) and sacrificed after 2 h. Species-specific secondary antibodies (S4 Table) were used to visualize specific taste cell markers and GLI3.

For antibody staining of organoids, cultured organoids were collected in 1.5 mL Eppendorf tubes and fixed for 15 min in fresh 4% paraformaldehyde in 1× PBS supplemented with MgCl_2_ (5 mM), EGTA (10 mM), and sucrose (4%, wt/v), washed three times for 5 min with 1× PBS, and blocked for 45 min with SuperBlock blocking buffer (Thermo Fisher cat. no. 37515) supplemented with 0.3% (v/v) Triton X-100 and 2% (v/v) donkey serum. They were then incubated at 4°C overnight with the desired primary antibodies (S4 Table). They were washed 3× for 5 min with 1× PBS and incubated for 1 h with species-specific secondary antibodies (1:500). 6-Diamidino-2-phenylindole (DAPI, 1:1000) in deionized water was used to visualize the nuclei following secondary antibody.

### Imaging

Bright-field images were generated using a Nikon DXM 1200C digital camera attached to a Nikon Eclipse 80i microscope and captured using Nikon NIS-Element F 3.00 software. Acquisition parameters were held constant for images with both antisense and sense probes. Fluorescent images were captured with the TCS SP2 Spectral Confocal Microscope (Leica Microsystems Wetzlar, Germany) using UV, Ar, GeNe, and HeNe lasers and appropriate excitation spectra. Scanware software (Leica Microsystems) was used to acquire z-series stacks captured at a step size of 2–3 μm. Acquisition parameters (i.e., gain, offset, PMT settings) were held constant for experiments with antibodies and for controls without antibodies. Digital images were cropped and arranged using Photoshop CS (Adobe Systems). Fluorescence images within a figure were adjusted for brightness and contrast for background standardization.

### Counting taste cells and taste buds

Quantitative measurements were carried out to determine the percentage of singly and doubly labeled type II and type III taste cells that co-expressed GLI3 and taste marker proteins. Confocal images from two to four sections from CV and FO papillae in each mouse were used for counting. To avoid counting the same cells more than once, sections separated from each other by at least 40 μm were chosen. Nuclear staining with DAPI was used to help distinguish individual taste cells. Only cells with entire cell bodies and nuclei visible were used for counting. GLI3-positive and taste-marker-labeled taste cells were counted in respective single-channel images, and the double-positive cells were counted using overlaid images.

KCNQ1 antibody staining was used to visualize taste buds for determination of taste bud size and taste cell number. Measurement of taste bud size was conducted as previously described [70]. Five *Gli3*^*CKO*^ and *Gli3*^*WT*^ mice were used for counting taste cell number. We found, on average, 10 taste buds per trench and 20 cells in each taste bud section. Only taste buds with typical morphology (with clear taste pore and the base of taste bud reaching the basement membrane) were used for analysis. Serial sections from similar regions of the tissue from each mouse were used to minimize location difference in taste bud number and size. The average number of nuclei in each taste bud was used as a proxy for the number of taste cells. For measuring the number of taste buds, all KCNQ1+ taste buds were counted, regardless of the morphology of taste bud. Double immunostaining was conducted using KCNQ1 antibody and respective taste cell marker antibodies to quantify the number of T1R3+, TRPM5+, GNAT3+, PKD2L1+, and CAR4+ taste cells per total taste cells per section. To quantify the percentage of taste cell subtypes in taste organoids with a clear single organoid profile from *Gli3*^*CKO*^ mice, single and double immunostaining was performed using specific taste cell markers or Gli3 antibody. Nuclear staining with DAPI was used to help distinguish individual taste cells. Only cells with entire cell bodies and nuclei visible were used for counting.

### Brief-access tests

Brief access tests were conducted using the Davis MS-160 mouse gustometer (Dilog Instruments, Tallahassee, FL) as described[71]. The following taste compounds were tested: sucrose (30, 100, 300, 1000 mM), sucralose (1, 3, 10, 30 mM), monosodium glutamate (MSG; 30, 100, 300, 100 mM), denatonium (0.3, 1, 3, 10 mM), citric acid (1, 3, 10, 30 mM), and NaCl (30, 100, 300, 600 mM). Mice were water- and food-restricted (1 g food and 1.5 mL water) for 23.5 h before test sessions for appetitive taste compounds (sucrose, sucralose, and MSG). For the aversive taste compounds (citric acid, denatonium, and NaCl), mice were water-deprived for 22.5 h before testing. In each test session, four different concentrations of each taste compound and water control were presented in a random order for 5 s after first lick, and the shutter reopened after a 7.5-s interval. The total test session time was 20 min. An additional 1-s “washout period” with water was interposed between each trial in sessions testing aversive tastants. *Gli3*^*CKO*^ and *Gli3*^*WT*^ mice were tested at the same time in parallel. Each mouse was tested with all the compounds. After each session mice were allowed to recover for 48 h with free access to food and water. Body weight of the mice was monitored daily, and only mice at or over 85% their initial body weight were used. The ratio of taste stimulus to water licks was calculated by dividing the number of licks for taste compounds by the number of licks for water presented in the parallel test session. Lick ratios > 1 indicate preference behavior to the taste compound, and lick ratios < 1 indicate avoidance behavior to the taste compound. With bitter and sour stimuli it appears that lick responses show a ceiling effect (maximum aversion) at higher concentrations. Thus, strain differences could only be seen at the lower concentrations.

### Gustatory nerve recording

The same sets of mice used for behavioral tests were used for electrophysiological recording of taste responses. Whole-nerve responses to tastants were recorded from the chorda tympani (CT) or the glossopharyngeal (GL) nerves as described [72]. Mice were anesthetized by an intraperitoneal injection (10 ml/kg, with 2.5 ml/kg further doses as necessary) of a mixture of ketamine (4.28 mg/ml), xylazine (0.86 mg/ml), and acepromazine (0.14 mg/ml). Under anesthesia, the trachea of each mouse was cannulated, and the mouse was then fixed in the supine position with a head holder to allow dissection of the CT or the GL nerve. The right CT nerve was dissected free from surrounding tissues after removal of the pterygoid muscle and cut at the point of its entry to the tympanic bulla. The right GL nerve from a different animal was exposed, dissected free from underlying tissues and cut near its entrance to the posterior lacerated foramen. All chemicals were used at ~24°C. The entire nerve was placed on the Ag-AgCl electrode. An indifferent electrode was placed in nearby tissue. For taste stimulation of fungiform papillae (FP), the anterior half of the tongue was enclosed in a flow chamber made of silicone rubber. For taste stimulation of the CV, an incision was made on each side of the animal's face from the corner of the mouth to just above the angle of the jaw, and the papillae were exposed and their trenches opened by slight tension applied through a small suture sewn in the tip of the tongue. For taste stimulation of fungiform papillae (FP), the anterior half of the tongue was enclosed in a flow chamber made of silicone rubber. Taste solutions were delivered to each part of the tongue by gravity flow for 30 s (CT) or 60 s (GL) at the same flow rate as the distilled water used for rinse (~0.1 ml/s). The following taste compounds were tested: sucrose (100, 300, 1000 mM), sucralose (3, 10, 30 mM), MSG (30, 100, 300 mM), denatonium (1, 3, 10 mM), citric acid (3, 10, 30 mM), and NaCl (30, 100, 300 mM). Neural responses resulting from chemical stimulations of the tongue were fed into an amplifier (K-1; Iyodenshikagaku, Nagoya, Japan) and monitored on an oscilloscope and an audio monitor. The whole-nerve responses were integrated with a time constant of 1.0 s, recorded using software (PowerLab 4/30; AD Instruments, Bella Vista, Australia), and analyzed using LabChart Pro software (AD Instruments). Nerve response magnitudes were measured at 5, 10, 15, 20, and 25 s after stimulus onset for the CT nerve and at 5, 10, 20, 30, and 40 s for the GL nerve. The stability of each preparation was monitored by the periodic application of 0.1 M NH_4_Cl. A recording was considered to be stable when the 0.1 M NH_4_Cl response magnitudes at the beginning and end of each stimulation series deviated by no more than 15%. Only responses from stable recordings were used for data analysis. At the end of the experiment, animals were killed by injecting an overdose of the anesthetic. The response values were averaged and normalized to responses to 100 mM NH_4_Cl to account for mouse-to-mouse variations in absolute responses. In Glossopharyngeal nerve recordings (S7 Fig), the responses appear not to return to baseline for some of the highest concentrations of stimuli because it is difficult and takes much time to wash them out completely from the CV cleft. Subsequent recordings were only done after repeated washings to make sure the previous response return to baseline. All data were compared as normalized units.

### Statistical analyses

Prism (GraphPad Software) was used for statistical analyses, including calculation of mean values, standard errors, and unpaired t-tests of cell counts and qPCR data. Data from taste behavioral tests and gustatory nerve recording were compiled using Microsoft Excel. For statistical analyses of behavioral and nerve responses, two-way ANOVA and post hoc t-tests were used to evaluate the difference between genotype (*Gli3*^*CKO*^ and *Gli3*^*WT*^ mice) and concentration using OriginPro (OriginLab). *p*-Values < 0.05 were considered significant.

## Supporting information

S1 Fig FACS and histochemistry controls. (A) Representative FACS plots of taste cells from wild-type, Lgr5-EGFP, Tas1r3-GFP, and GAD-1-GFP transgenic mice. Sorted GFP fluorescent cells are shown in the boxed regions. (B-E) In situ hybridization using digoxigenin-labeled Gli3 RNA probes in jejunum (positive control) (B-D) and circumvallate papilla (CV) from a Skn-1a knockout (KO) mice (negative control) (E). C is higher magnification of the boxed area in B. Signal from Gli3 sense probe in jejunum indicative of nonspecific background was lower than with the antisense probe, and no signal was produced in CV of Skn-1a knockout mouse, which lacks all type II cells. (F-G) GLI3 was detected by immunostaining in jejunum (H) but not in the CV taste cells of Skn-1a knockout mice. G is higher magnification of the boxed area in F. Omission of the primary antibody demonstrates low nonspecific background from secondary antibody in wild-type (WT) CV (I). Scale bars: B-D, F and G: 100 μm; E, H, and I: 50 μm.
(TIF)

S2 Fig GLI3 is not expressed in type I and type III taste receptor cells. Double-labeled indirect immunofluorescence confocal microscopy of fungiform (FF; A, D, G), folate (FO; B, E, H), and circumvallate (CV; C, F, I) papillae sections stained with antibodies against GLI3 and the type III taste cell marker CAR4 (A-C), serotonin (5-HT) (D-F) or type I cells marked by intrinsic GFP fluorescence in *Glast1*-GFP transgenic mice (G-I). Merged images show lack of co-expression of GLI3 with the type I or type III markers. Scale bars, 100 μm.
(TIF)

S3 Fig Effect of *Gli3* deficiency on type III taste cells. (A-H) Indirect immunofluorescence confocal microscopy of circumvallate (CV) sections from 5 *Gli3*^*WT*^ (A-C) and 5 *Gli3*^*CKO*^ (E-G) mice stained with antibodies against PKD2L1 (A, E), CAR4 (B, F) and GLI3 (C, G). Nuclei were counterstained with DAPI (blue). (D, H) GFP expression from the *Gli3* knockin is turned on by Cre-mediated excision of *Gli3*. (I) Cell counting shows that the percentage of PKD2L1-labeled type III cells (t = 1.43, p>0.05) among total taste receptor cells is unchanged, but that of CAR4- (t = 3.31, p<0.01) and GLI3-labeled cells (t = 14.72, p<0.0001) decreased in *Gli3*^*CKO*^ mice. (J) qPCR showed that the expression of *Pkd2l1*, *Car4* and *Snap25* mRNAs remained unchanged while that of *Gli3* and *NTPDase2* decreased in CV papillae taste cells from *Gli3*^*CKO*^ mice compared to those of *Gli3*^*WT*^ mice. Data are means + SEM. **p<0.01, ****p<0.0001.
(TIF)

S4 Fig Effect of *Gli3* deficiency on taste bud size and composition in foliate papillae. (A) Composite confocal image of Lgr5-EGFP+ cells (green) in FO papillae sections from an *Lgr5-EGFP-ires-CreERT2+/-* mouse. (B-O) Indirect immunofluorescence confocal microscopy of FO sections from 5 control and 5 *Gli3* conditional knockout (*Gli3*^*CKO*^) mice immunostained with antibodies against: KCNQ1 to label all taste cells (B, C); against TRPM5 (D, E), T1R3 (F, G), and GNAT3 (H, I) to label type II taste cells; against PKD2L1 (J, K) and CAR4 (L, M) to label type III taste cells; and against GLI3 (N, O) to confirm *Gli3* gene deletion. Nuclei are counterstained with DAPI (blue). Scale bars indicate 100 μm. (P) Compared to control (*Gli3*^*WT*^) mice, the average number of cells in taste buds (t = 3.12, p<0.01) and the size (in μm^2^) of the taste buds (t = 4.91, p<0.0001) increased in FO papillae from *Gli3*^*CKO*^ mice. (Q) Cell counting in *Gli3*^*CKO*^ mice shows that the proportion of TRPM5- (t = 4.34, p<0.05) and T1R3- (t = 5.87, p<0.0001) but not GNAT3-labeled type II taste receptors cells (t = 0.42, p>0.05) or PKD2L1- (t = 0.44, p>0.05) and CAR4-labeled type III cells (t = 0.19, p>0.05) increased, while the proportion of GLI3-labeled cells decreased dramatically. Five control and *Gli3*^*CKO*^ mice each were used for analyses. Data are means + SEM. **p<0.01, ****p<0.0001.
(TIF)

S5 Fig Gli3 deficiency increases taste stem cell abundance and affects *Lgr5* and Shh target gene expression. (A) Representative FACS plots of taste cells from *Gli3*^*CKO*^ and *Gli3*^*WT*^ mice show an increase in the proportion of Lgr5-GFP cells (bracketed area) in *Gli3*^*CKO*^ mice. (n = 5) (B-D) qPCR shows increased expression of *Lgr5* mRNA in FACS-purified Lgr5-GFP taste cells (t = 4.14, p<0.05) (B) and in CV papillae from *Gli3*^*CKO*^ mice (t = 3.58, p<0.05) (C). As expected, *Gli3* expression in FACS-purified Lgr5-GFP cells was markedly reduced (t = 12.77, p<0.0001) (B). The expression of the *Gli3* target genes *Gli1 Ccnd2*, and *Mycn* did not change significantly, while that of the target gene *Jag2 (t = 2*.*88*, *p<0*.*05)* decreased in CV papillae from *Gli3*^*CKO*^ mice. Among the upstream regulators of *Gli3*, expression of *Ptch1 (t = 9*.*00*, *p<0*.*001)* increased while that of *Smo* did not change significantly (D). Data are means + SEM. *p<0.05, **p<0.01, ***p<0.001, p****<0.0001.
(TIF)

S6 Fig *Gli3* deficiency affects taste cell differentiation and expression of Shh pathway target genes *ex vivo*. (A-H) Indirect immunofluorescence confocal microscopy of taste organoids cultured from individual FACS-sorted Lgr5-GFP cells isolated from CV papillae of 5 double-knockin mice treated with tamoxifen (*Gli3*^*CKO*^) (B, D, F) or untreated (*Gli3*^*WT*^) (A, C, E), then stained with antibodies against CAR4 (A, B), NTPDase2 (C, D), or GLI3 (E, F). (G, H) The intrinsic GFP fluorescence in *Gli3*^*CKO*^ organoids shows that GFP expression is turned on following *Gli3* deletion. Scale bars, 100 μm. (I) The number of CAR4+ (n = 90, t = 2.84, p<0.05) and GLI3+ (n = 96, t = 13.27, p<0.0001) cells decreased significantly in *Gli3*^*CKO*^ vs. *Gli3*^*WT*^ organoids. (J, K) qPCR showed that expression of several taste cell type specific marker genes [*Pkd2l1 (t = 3*.*17*, *p<0*.*05)*, *Snap25 (t = 4*.*54*, *p<0*.*01)*, *Gli3 (t = 11*.*58*, *p<0*.*001)*] and Shh pathway target genes [*Gli1 (t = 3*.*89*, *p<0*.*05)*, *Ccnd2 (t = 3*.*97*, *p<0*.*05)*, *Mycn (t = 3*.*12*, *p<0*.*05)*, and *Jag2 (t = 2*.*87*, *p<0*.*05)*] decreased, while that of *Lgr5* (t = 3.18, p<0.05) and the Shh receptor *Ptch1(t = 4*.*45*, *p<0*.*01)* increased in *Gli3*^*CKO*^ organoids relative to those from *Gli3*^*WT*^ mice. Data are means + SEM. *p<0.05, **p<0.01, ****p<0.0001.
(TIFF)

S7 Fig Sample recordings of integrated whole nerve responses from the glossopharyngeal nerve to increasing concentrations of tastants in *Gli3*^*WT*^ and *Gli3*^*CKO*^ mice. (A) Exemplars of continuous recordings of GL nerve responses to multiple tastants in *Gli3*^*WT*^ and *Gli3*^*CKO*^ mice. The response values were normalized to responses to 100mM NH_4_Cl bracketing the stimuli at beginning and end of the recording period. Abbreviations: Suc, sucrose; Sucra, sucralose; DB, denatonium benzoate; MSG, monosodium glutamate; NaCl, Sodium chloride; NH_4_Cl, Ammonium chloride. (B) Exemplar traces of responses to indicated taste stimuli. Shaded boxes indicate the response in *Gli3*^*WT*^ (blue) and the increase in response in *Gli3*^*CKO*^ above that in *Gli3*^*WT*^ mice (pink). All recordings shown are cut from continuous recordings from the same *Gli3*^*WT*^ or *Gli3*^*CKO*^ animal. Some responses to do not return to baseline immediately after the end of stimulation, but subsequent recordings were done only after repeated washout of stimuli to ensure the responses did indeed return to baseline (see Methods). Horizontal bars at the bottom of the traces in A and B indicate duration of taste stimulation (60 sec).
(TIF)

S8 Fig *Gli3*^*CKO*^ mice display unchanged chorda tympani (CT) nerve responses to almost all taste stimuli. Sample recordings of integrated nerve responses to tastants (blue boxes). (A) Exemplars of continuous recordings of integrated whole nerve responses from the chorda tympani (CT) nerve to indicated tastants in *Gli3*^*WT*^ and *Gli3*^*CKO*^ mice. Abbreviations: Suc, sucrose; DB, denatonium benzoate; MSG, monosodium glutamate; NaCl, Sodium chloride; NH_4_Cl, Ammonium chloride. The response values were normalized to responses to 100mM NH_4_Cl bracketing the other stimuli at beginning and end of the recording period. (B) Average CT nerve responses to sweet (sucrose and sucralose, C,D), umami (MSG), E), bitter (denatonium, F), salty (NaCl, G), and sour (citric acid, H) tasting compounds are shown. All recordings shown are cut from continuous recordings from the same Gli3^WT^ or Gli3^CKO^ animal. *Gli3*^*CKO*^ and *Gli3*^*WT*^ mice showed comparable CT nerve responses to all taste compounds tested, except to some concentrations of sucralose and citric acid. Data are means ± SEM. Statistically significant differences were determined by repeated two-way ANOVA test [sucrose: F (1, 32) = 3.76, P>0.05; sucralose: F (1, 24) = 21.58, P<0.05; MSG: F (1, 29) = 0.05, P>0.05; denatonium: F (1, 29) = 1.77, P>0.05; NaCl: F (1, 23) = 3.80, P>0.05; citric acid: F(1, 26) = 4.20, P<0.05] and post hoc t-test (n≥4, *p<0.05, **p<0.05). Horizontal bars at the bottom of the traces in A and B indicate duration of taste stimuli (30 sec). (C-H): n≥5 for each genotype.
(TIF)

S1 Table Expression of Shh pathway components in single-cell RNA-Seq data from Lgr5-GFP+, Gad1-GFP+, and Tas1r3-GFP+ taste cells. The normalized counts and false discovery rate (FDR) of the indicated pairwise comparison in each single-cell library is shown.
(XLSX)

S2 Table Co-expression of GLI3 with taste marker genes. Mouse taste cells from CV and FO papillae were doubly stained for GLI3 and taste cell markers CAR4, 5-HT or TRPM5, or singly stained for GLI3 in sections from Tas1r3-GFP, TRPM5, Gnat3-GFP or Glast1-GFP transgenic mice. Singly and doubly labeled cells were counted to determine co-expression. Numerators are the numbers of taste cells expressing both gene 1 and gene 2. Denominators are the numbers of taste cells expressing gene 1. Taste cells expressing both gene 1 and gene 2 as a percentage of those expressing gene 1 are shown in parentheses. ND, not determined.
(DOCX)

S3 Table Sequence of primers used for RT-PCR and qPCR. (DOCX)

S4 Table List of antibodies and their concentrations used in this study. (DOCX)
