# Supplementary figures and images for: Gli3 is a negative regulator of Tas1r3-expressing taste cells
Source: PLoS Genet. 2018 Feb 7;14(2):e1007058. doi: 10.1371/journal.pgen.1007058 (PMC5819828; doi:10.1371/journal.pgen.1007058)

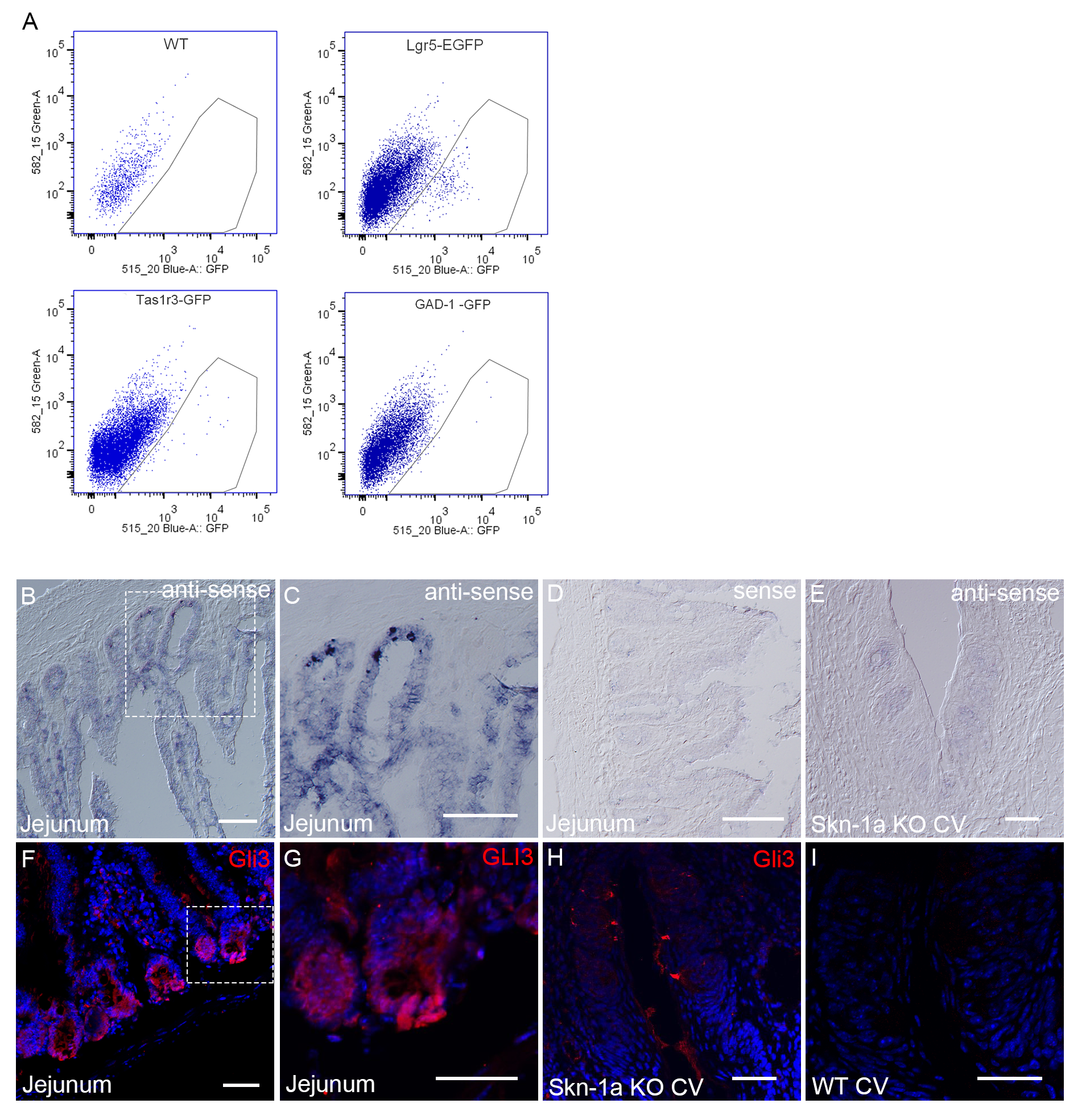

Supplement: S1 Fig — (A) Representative FACS plots of taste cells from wild-type, Lgr5-EGFP, Tas1r3-GFP, and GAD-1-GFP transgenic mice. Sorted GFP fluorescent cells are shown in the boxed regions. (B-E) In situ hybridization using digoxigenin-labeled Gli3 RNA probes in jejunum (positive control) (B-D) and circumvallate papilla (CV) from a Skn-1a knockout (KO) mice (negative control) (E). C is higher magnification of the boxed area in B. Signal from Gli3 sense probe in jejunum indicative of nonspecific background was lower than with the antisense probe, and no signal was produced in CV of Skn-1a knockout mouse, which lacks all type II cells. (F-G) GLI3 was detected by immunostaining in jejunum (H) but not in the CV taste cells of Skn-1a knockout mice. G is higher magnification of the boxed area in F. Omission of the primary antibody demonstrates low nonspecific background from secondary antibody in wild-type (WT) CV (I). Scale bars: B-D, F and G: 100 μm; E, H, and I: 50 μm. (TIF) [file pgen.1007058.s001.tif]

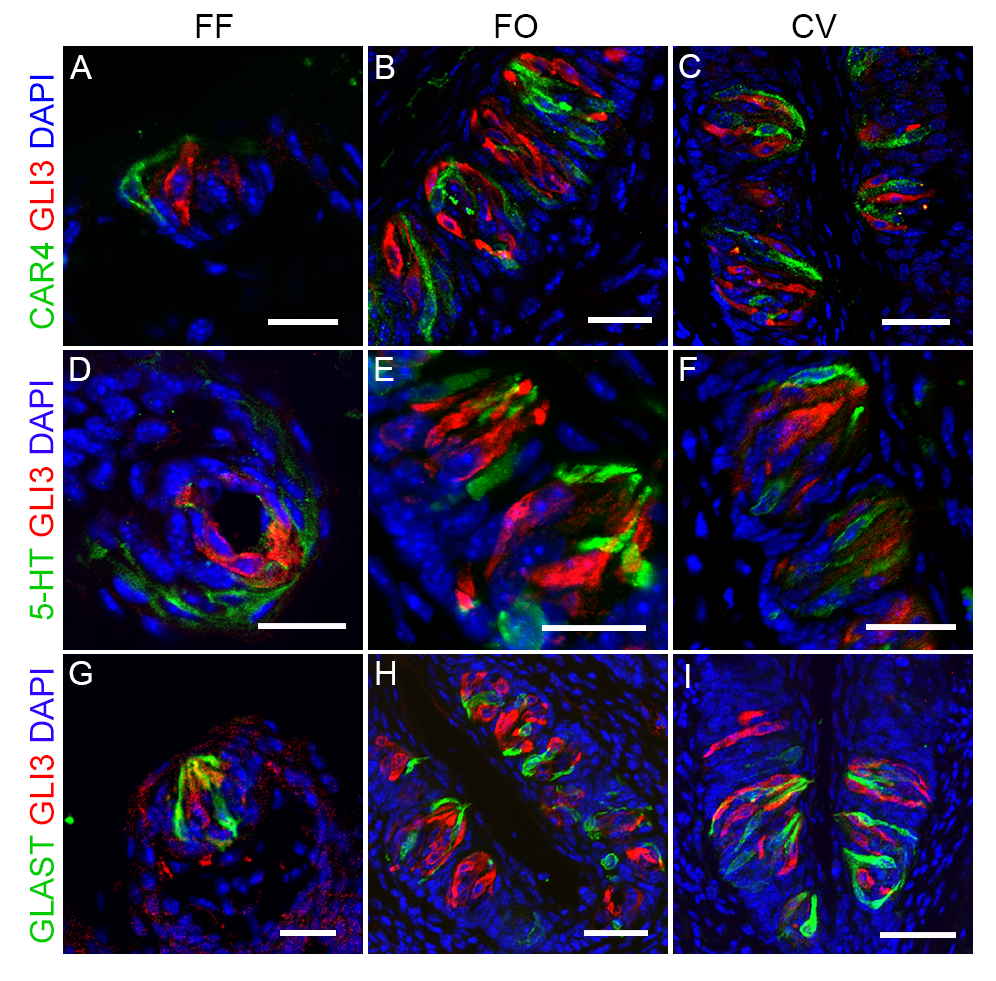

Supplement: S2 Fig — Double-labeled indirect immunofluorescence confocal microscopy of fungiform (FF; A, D, G), folate (FO; B, E, H), and circumvallate (CV; C, F, I) papillae sections stained with antibodies against GLI3 and the type III taste cell marker CAR4 (A-C), serotonin (5-HT) (D-F) or type I cells marked by intrinsic GFP fluorescence in Glast1-GFP transgenic mice (G-I). Merged images show lack of co-expression of GLI3 with the type I or type III markers. Scale bars, 100 μm. (TIF) [file pgen.1007058.s002.tif]

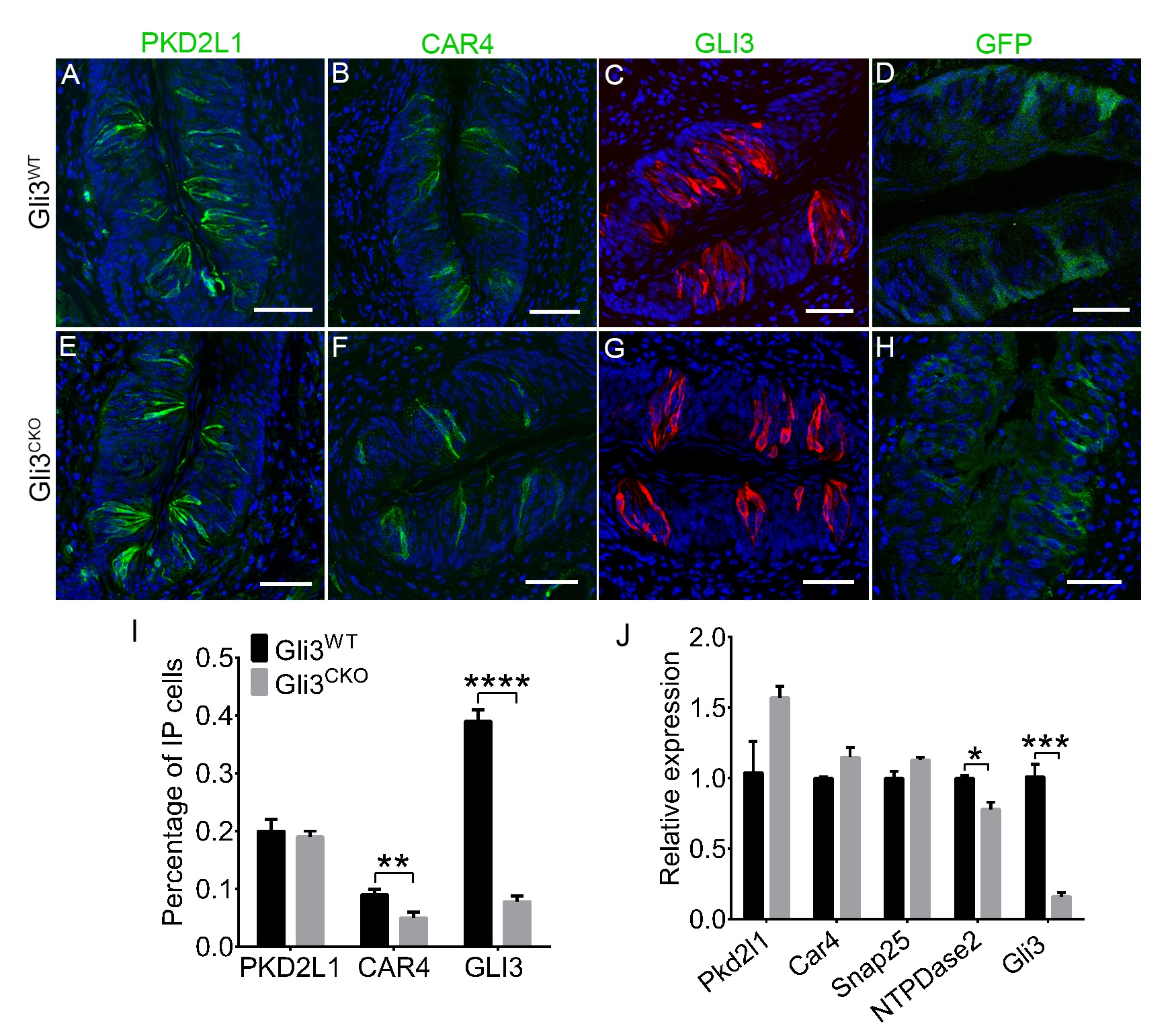

Supplement: S3 Fig — (A-H) Indirect immunofluorescence confocal microscopy of circumvallate (CV) sections from 5 Gli3WT (A-C) and 5 Gli3CKO (E-G) mice stained with antibodies against PKD2L1 (A, E), CAR4 (B, F) and GLI3 (C, G). Nuclei were counterstained with DAPI (blue). (D, H) GFP expression from the Gli3 knockin is turned on by Cre-mediated excision of Gli3. (I) Cell counting shows that the percentage of PKD2L1-labeled type III cells (t = 1.43, p>0.05) among total taste receptor cells is unchanged, but that of CAR4- (t = 3.31, p<0.01) and GLI3-labeled cells (t = 14.72, p<0.0001) decreased in Gli3CKO mice. (J) qPCR showed that the expression of Pkd2l1, Car4 and Snap25 mRNAs remained unchanged while that of Gli3 and NTPDase2 decreased in CV papillae taste cells from Gli3CKO mice compared to those of Gli3WT mice. Data are means + SEM. **p<0.01, ****p<0.0001. (TIF) [file pgen.1007058.s003.tif]

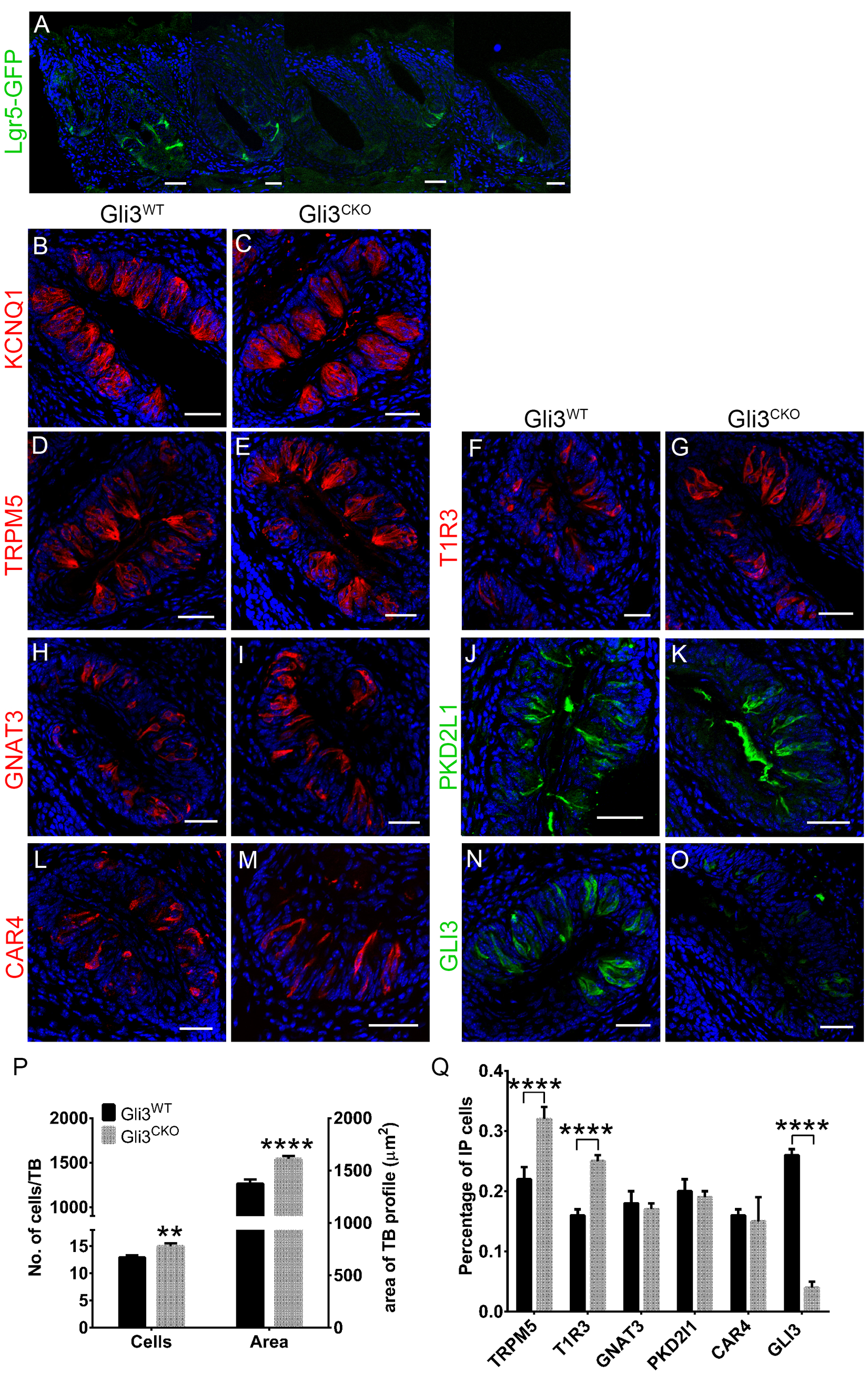

Supplement: S4 Fig — (A) Composite confocal image of Lgr5-EGFP+ cells (green) in FO papillae sections from an Lgr5-EGFP-ires-CreERT2+/- mouse. (B-O) Indirect immunofluorescence confocal microscopy of FO sections from 5 control and 5 Gli3 conditional knockout (Gli3CKO) mice immunostained with antibodies against: KCNQ1 to label all taste cells (B, C); against TRPM5 (D, E), T1R3 (F, G), and GNAT3 (H, I) to label type II taste cells; against PKD2L1 (J, K) and CAR4 (L, M) to label type III taste cells; and against GLI3 (N, O) to confirm Gli3 gene deletion. Nuclei are counterstained with DAPI (blue). Scale bars indicate 100 μm. (P) Compared to control (Gli3WT) mice, the average number of cells in taste buds (t = 3.12, p<0.01) and the size (in μm2) of the taste buds (t = 4.91, p<0.0001) increased in FO papillae from Gli3CKO mice. (Q) Cell counting in Gli3CKO mice shows that the proportion of TRPM5- (t = 4.34, p<0.05) and T1R3- (t = 5.87, p<0.0001) but not GNAT3-labeled type II taste receptors cells (t = 0.42, p>0.05) or PKD2L1- (t = 0.44, p>0.05) and CAR4-labeled type III cells (t = 0.19, p>0.05) increased, while the proportion of GLI3-labeled cells decreased dramatically. Five control and Gli3CKO mice each were used for analyses. Data are means + SEM. **p<0.01, ****p<0.0001. (TIF) [file pgen.1007058.s004.tif]

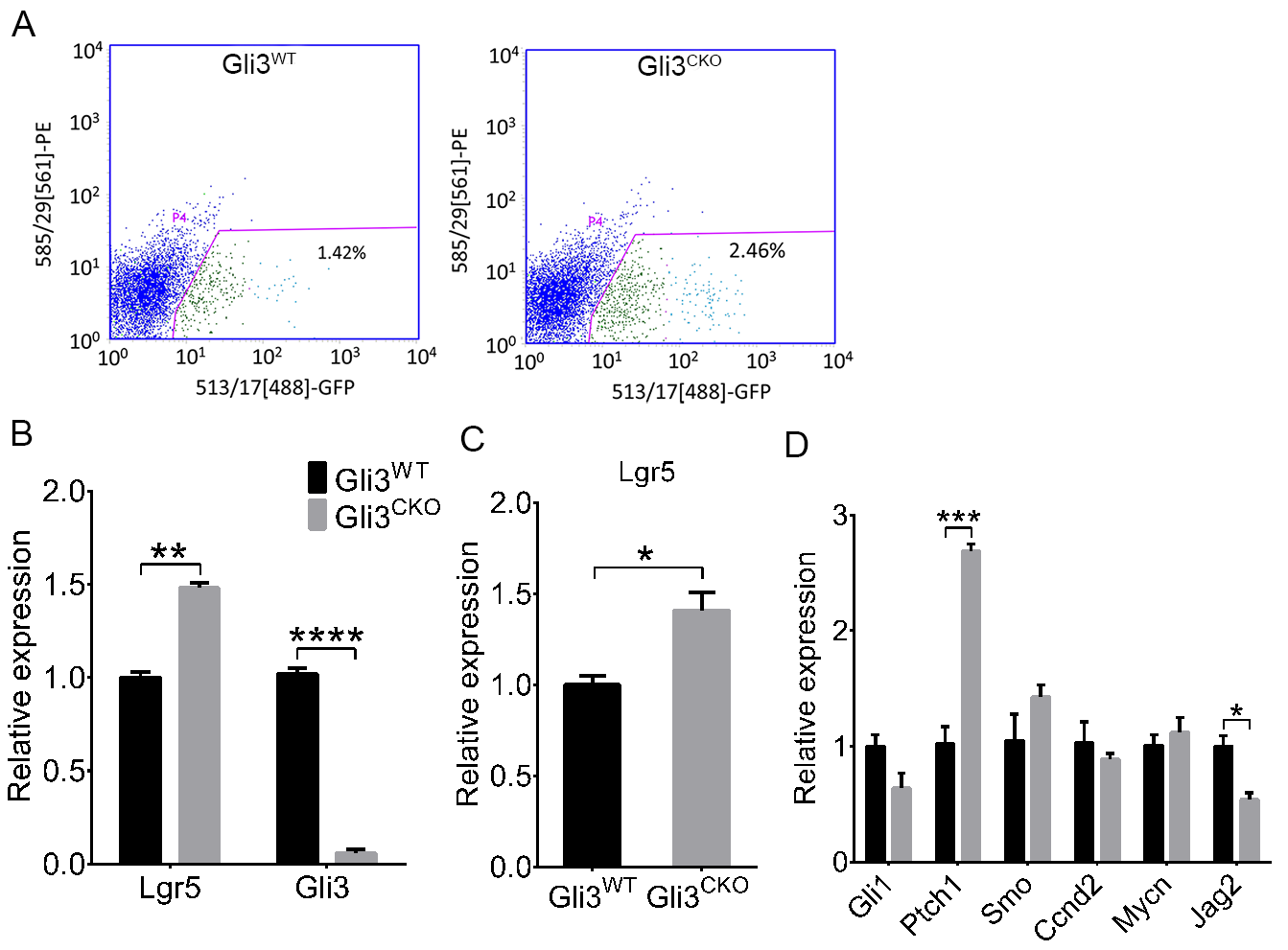

Supplement: S5 Fig — (A) Representative FACS plots of taste cells from Gli3CKO and Gli3WT mice show an increase in the proportion of Lgr5-GFP cells (bracketed area) in Gli3CKO mice. (n = 5) (B-D) qPCR shows increased expression of Lgr5 mRNA in FACS-purified Lgr5-GFP taste cells (t = 4.14, p<0.05) (B) and in CV papillae from Gli3CKO mice (t = 3.58, p<0.05) (C). As expected, Gli3 expression in FACS-purified Lgr5-GFP cells was markedly reduced (t = 12.77, p<0.0001) (B). The expression of the Gli3 target genes Gli1 Ccnd2, and Mycn did not change significantly, while that of the target gene Jag2 (t = 2.88, p<0.05) decreased in CV papillae from Gli3CKO mice. Among the upstream regulators of Gli3, expression of Ptch1 (t = 9.00, p<0.001) increased while that of Smo did not change significantly (D). Data are means + SEM. *p<0.05, **p<0.01, ***p<0.001, p****<0.0001. (TIF) [file pgen.1007058.s005.tif]

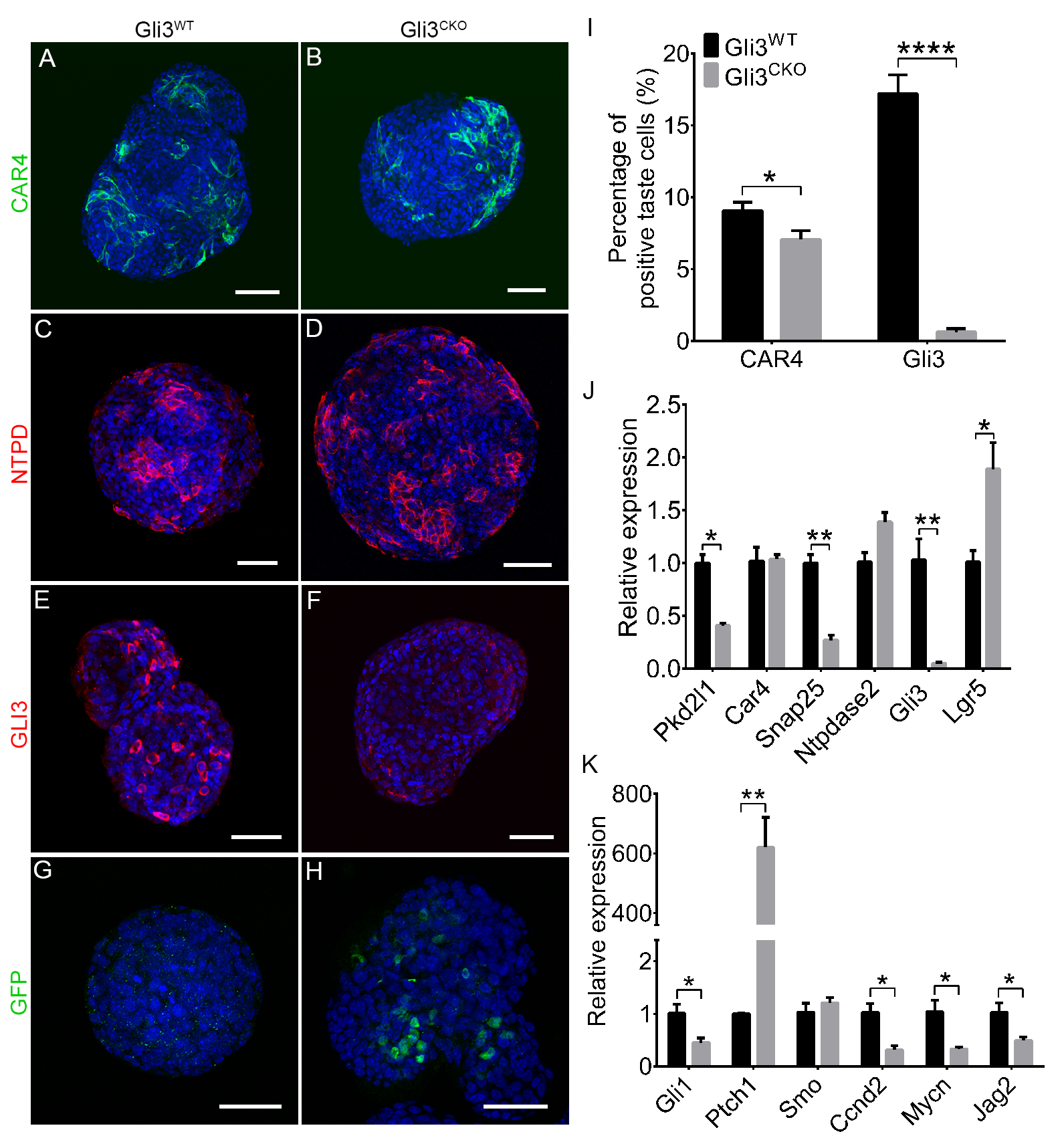

Supplement: S6 Fig — (A-H) Indirect immunofluorescence confocal microscopy of taste organoids cultured from individual FACS-sorted Lgr5-GFP cells isolated from CV papillae of 5 double-knockin mice treated with tamoxifen (Gli3CKO) (B, D, F) or untreated (Gli3WT) (A, C, E), then stained with antibodies against CAR4 (A, B), NTPDase2 (C, D), or GLI3 (E, F). (G, H) The intrinsic GFP fluorescence in Gli3CKO organoids shows that GFP expression is turned on following Gli3 deletion. Scale bars, 100 μm. (I) The number of CAR4+ (n = 90, t = 2.84, p<0.05) and GLI3+ (n = 96, t = 13.27, p<0.0001) cells decreased significantly in Gli3CKO vs. Gli3WT organoids. (J, K) qPCR showed that expression of several taste cell type specific marker genes [Pkd2l1 (t = 3.17, p<0.05), Snap25 (t = 4.54, p<0.01), Gli3 (t = 11.58, p<0.001)] and Shh pathway target genes [Gli1 (t = 3.89, p<0.05), Ccnd2 (t = 3.97, p<0.05), Mycn (t = 3.12, p<0.05), and Jag2 (t = 2.87, p<0.05)] decreased, while that of Lgr5 (t = 3.18, p<0.05) and the Shh receptor Ptch1(t = 4.45, p<0.01) increased in Gli3CKO organoids relative to those from Gli3WT mice. Data are means + SEM. *p<0.05, **p<0.01, ****p<0.0001. (TIFF) [file pgen.1007058.s006.tiff]

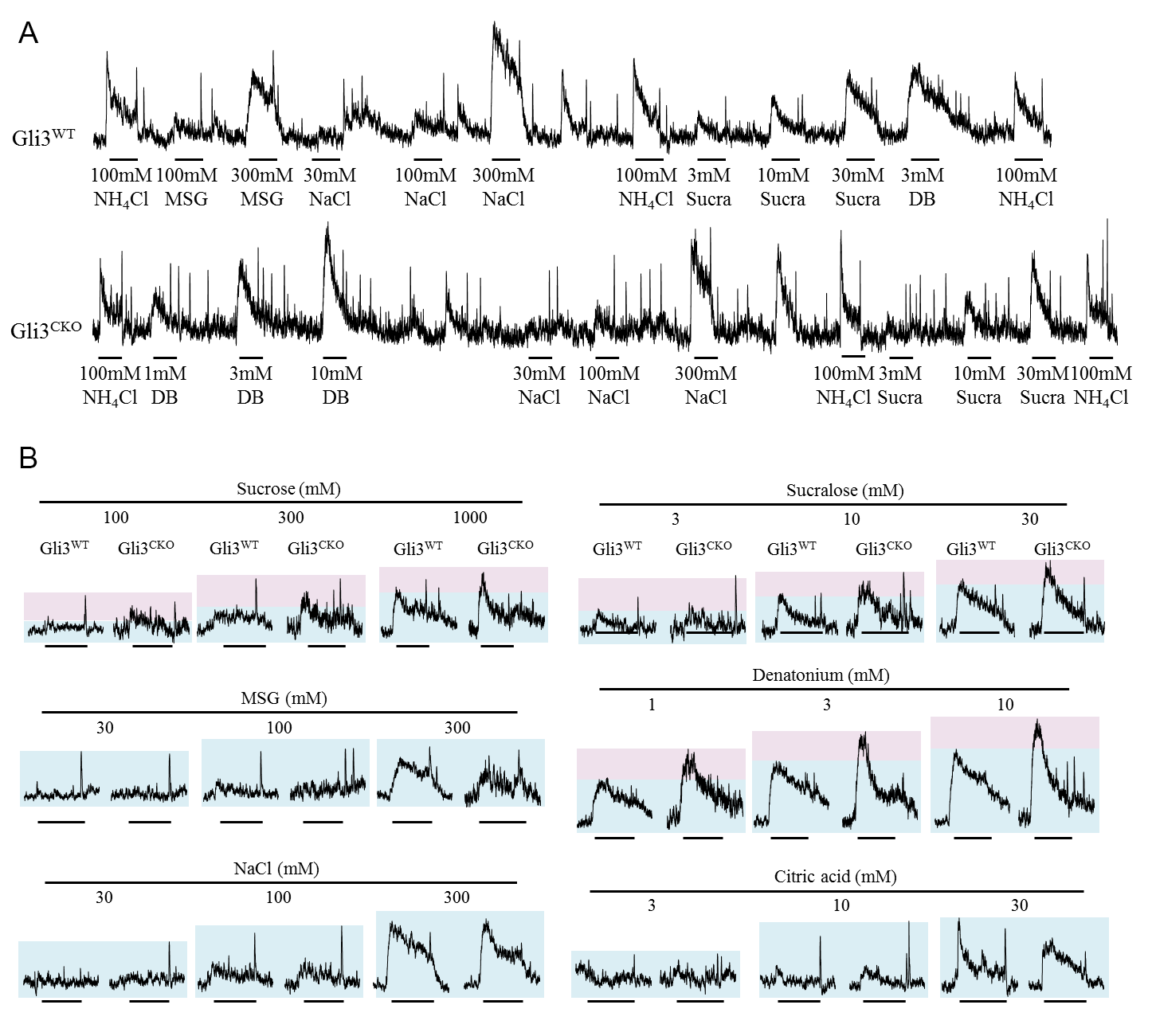

Supplement: S7 Fig — (A) Exemplars of continuous recordings of GL nerve responses to multiple tastants in Gli3WT and Gli3CKO mice. The response values were normalized to responses to 100mM NH4Cl bracketing the stimuli at beginning and end of the recording period. Abbreviations: Suc, sucrose; Sucra, sucralose; DB, denatonium benzoate; MSG, monosodium glutamate; NaCl, Sodium chloride; NH4Cl, Ammonium chloride. (B) Exemplar traces of responses to indicated taste stimuli. Shaded boxes indicate the response in Gli3WT (blue) and the increase in response in Gli3CKO above that in Gli3WT mice (pink). All recordings shown are cut from continuous recordings from the same Gli3WT or Gli3CKO animal. Some responses to do not return to baseline immediately after the end of stimulation, but subsequent recordings were done only after repeated washout of stimuli to ensure the responses did indeed return to baseline (see Methods). Horizontal bars at the bottom of the traces in A and B indicate duration of taste stimulation (60 sec). (TIF) [file pgen.1007058.s007.tif]

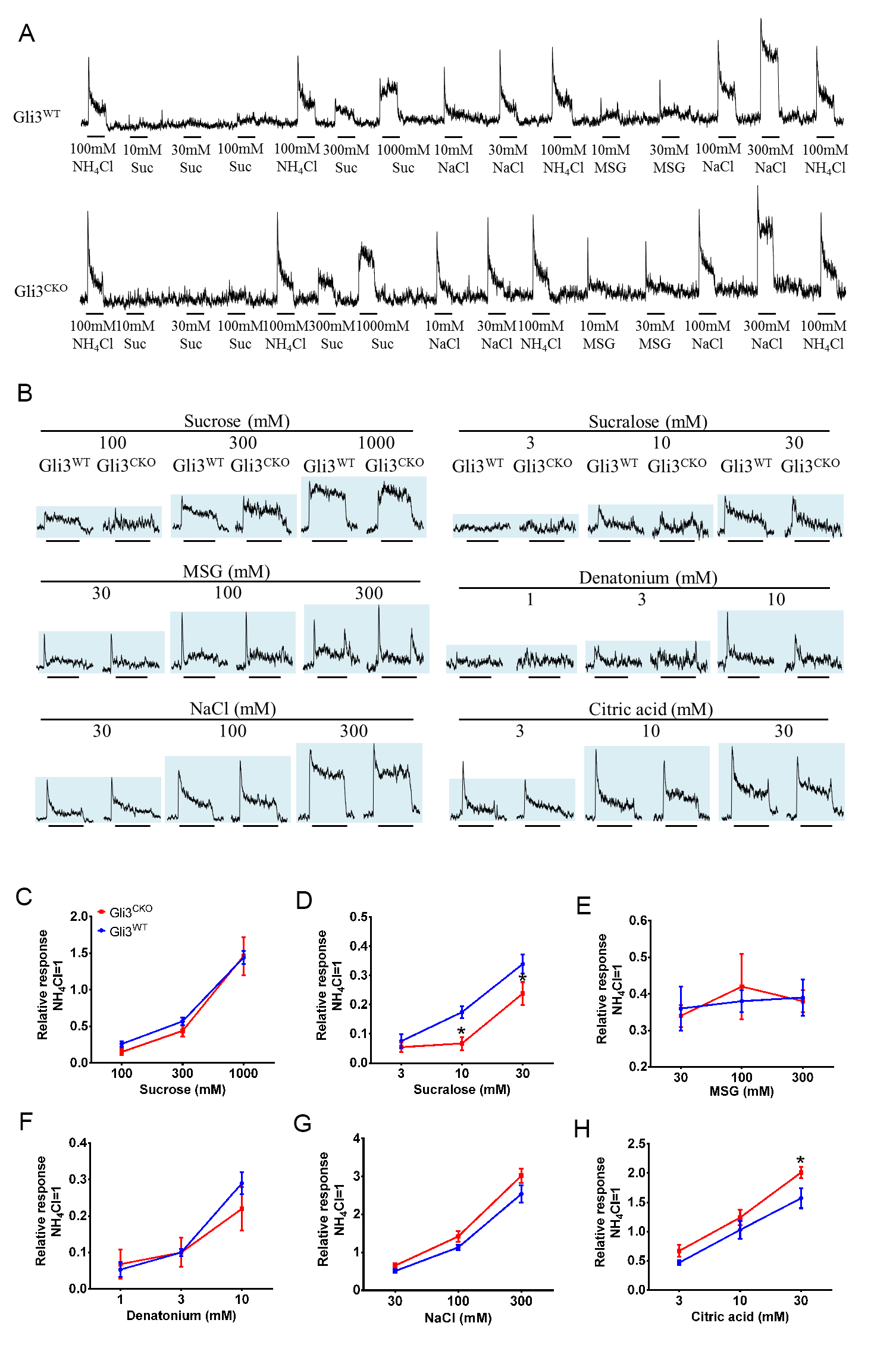

Supplement: S8 Fig — Sample recordings of integrated nerve responses to tastants (blue boxes). (A) Exemplars of continuous recordings of integrated whole nerve responses from the chorda tympani (CT) nerve to indicated tastants in Gli3WT and Gli3CKO mice. Abbreviations: Suc, sucrose; DB, denatonium benzoate; MSG, monosodium glutamate; NaCl, Sodium chloride; NH4Cl, Ammonium chloride. The response values were normalized to responses to 100mM NH4Cl bracketing the other stimuli at beginning and end of the recording period. (B) Average CT nerve responses to sweet (sucrose and sucralose, C,D), umami (MSG), E), bitter (denatonium, F), salty (NaCl, G), and sour (citric acid, H) tasting compounds are shown. All recordings shown are cut from continuous recordings from the same Gli3WT or Gli3CKO animal. Gli3CKO and Gli3WT mice showed comparable CT nerve responses to all taste compounds tested, except to some concentrations of sucralose and citric acid. Data are means ± SEM. Statistically significant differences were determined by repeated two-way ANOVA test [sucrose: F (1, 32) = 3.76, P>0.05; sucralose: F (1, 24) = 21.58, P<0.05; MSG: F (1, 29) = 0.05, P>0.05; denatonium: F (1, 29) = 1.77, P>0.05; NaCl: F (1, 23) = 3.80, P>0.05; citric acid: F(1, 26) = 4.20, P<0.05] and post hoc t-test (n≥4, *p<0.05, **p<0.05). Horizontal bars at the bottom of the traces in A and B indicate duration of taste stimuli (30 sec). (C-H): n≥5 for each genotype. (TIF) [file pgen.1007058.s008.tif]
