# Supplementary material for: Gli3 is a negative regulator of Tas1r3-expressing taste cells
Source: PLoS Genet. 2018 Feb 7;14(2):e1007058. doi: 10.1371/journal.pgen.1007058 (PMC5819828; doi:10.1371/journal.pgen.1007058)
Supplement: S2 Table — Mouse taste cells from CV and FO papillae were doubly stained for GLI3 and taste cell markers CAR4, 5-HT or TRPM5, or singly stained for GLI3 in sections from Tas1r3-GFP, TRPM5, Gnat3-GFP or Glast1-GFP transgenic mice. Singly and doubly labeled cells were counted to determine co-expression. Numerators are the numbers of taste cells expressing both gene 1 and gene 2. Denominators are the numbers of taste cells expressing gene 1. Taste cells expressing both gene 1 and gene 2 as a percentage of those expressing gene 1 are shown in parentheses. ND, not determined. (DOCX) [file pgen.1007058.s010.docx]

**Table S2. Co-expression of GLI3 with taste marker genes.**

| **Gene 1** | **Gene 2** | | | | | | |
| --- | --- | --- | --- | --- | --- | --- | --- |
|  | **GLI3** | **T1R3** | **TRPM5** | **GNAT3** | **CAR4** | **5-HT** | **GLAST** |
| Number of circumvallate taste cells expressing one or both genes | | | | | | | |
| **GLI3** | － | 86/87 (98.8%) | 66/68 (97.1%) | 37/104 (35.6%) | 4/152 (2.6%) | 1/92  (1.1%) | 0/86 (0%) |
| **T1R3** | 86/93 (92.5%) | － | ND | ND | ND | ND | ND |
| **TRPM5** | 66/103  (64.1%) | ND | － | ND | ND | ND | ND |
| **GNAT3** | 37/119 (31.1%) | ND | ND | － | ND | ND | ND |
| **CAR4** | 4/108  (3.7%) | ND | ND | ND | － | ND | ND |
| **5-HT** | 1/68  (1.4%) | ND | ND | ND | ND | － | ND |
| **GLAST** | 0/88  (0%) | ND | ND | ND | ND | ND | － |
| Number of foliate taste cells expressing one or both genes | | | | | | | |
| **GLI3** | － | 112/115 (97.4%) | 99/100 (99.0%) | 113/180 (62.8%) | 1/87 (2.6%) | 1/167 (0.6%) | 0/92 (0%) |
| **T1R3** | 112/121 (92.3%) | － | ND | ND | ND | ND | ND |
| **TRPM5** | 99/218 (45.4%) | ND | － | ND | ND | ND | ND |
| **GNAT3** | 113/221 (51.1%) | ND | ND | － | ND | ND | ND |
| **CAR4** | 1/44 (2.3%) | ND | ND | ND | － | ND | ND |
| **5-HT** | 1/99 (1.0%) | ND | ND | ND | ND | － | ND |
| **GLAST** | 0/64 (0%) | ND | ND | ND | ND | ND | － |

Mouse taste cells from CV and FO papillae were doubly stained for GLI3 and taste cell markers CAR4, 5-HT or TRPM5, or singly stained for GLI3 in sections from Tas1r3-GFP, TRPM5, Gnat3-GFP or Glast1-GFP transgenic mice. Singly and doubly labeled cells were counted to determine co-expression. Numerators are the numbers of taste cells expressing both gene 1 and gene 2. Denominators are the numbers of taste cells expressing gene 1 or gene 2. Taste cells expressing both gene 1 and gene 2 as a percentage of those expressing gene 1 or gene 2 are shown in parentheses. ND, not determined.
