# Supplementary material for: Gli3 is a negative regulator of Tas1r3-expressing taste cells
Source: PLoS Genet. 2018 Feb 7;14(2):e1007058. doi: 10.1371/journal.pgen.1007058 (PMC5819828; doi:10.1371/journal.pgen.1007058)
Supplement: S3 Table — (DOCX) [file pgen.1007058.s011.docx]

| **Gene name** | **Forward primer** | **Reverse primer** |
| --- | --- | --- |
| *Gli3* | GGGAAGAGTGCCTCCAGGTG | TGATGGGGGACTGTTGGCTG |
| *Tas1r3* | CAAGTTCTTCAGCTTCTTCC | GGCGGCCACCCAGTTCCAGC |
| *Trmp5* | GCAAATCCTCTCTGGATGAAA | TAGCTGAACATGGCGATCAG |
| *Gnat3* | CATGGCTACACTGGGGATTG | GATTTCAGCCAGCTGTGGAG |
| *Gna14* | ATCAGAGCAATGGATACCCTGAG | GAGAGTGCAGTGACCTTGTCT |
| *Pkd2l1* | TCTGGACCTGGTGGTCATCT | CCCATCAGTCGGTTCACTTC |
| *Snap25* | ACCTAGGAAAATTCTGCGGG | CTGGCCACTACTCCATCCTG |
| *Car4* | CAAGTTTGCAGTGCTGGCAT | GCAAACTGCTCTCCCTCACT |
| *Ntpdase2* | CATGCGCCTACTCAACCTGA | GTGTGAG CGTCTGTGTCACT |
| *Lgr5* | TAAAGACGACGGCAACAGTG | GATTCGGATCAGCCAGCTAC |
| *Gli1* | CTTCAAGGCCCAATACATGC | CTTGAGGTTTTCAAGGCGTG |
| *Ptch1* | CTAGCAATAGGGACCGCTCA | GTCTCAGGGTAGCTCTCATAGC |
| *Smo* | GGTGCTTATTGTGGGAGGCT | TTCTCACTCAGAAGCCCAGG |
| *Ccnd2* | GTGCAGAAGGACATCCAACC | TTCATGGCCAGAGGAAAGAC |
| *Mycn* | ACCCCTTTGGCTCATTCTCT | TATGGGGGAGTGCTTCCTTC |
| *Jag2* | TGGGACAATGACACCACTCC | TGAAGTGCAGGCTCTTCCAG |
| *Gapdh* | GCATGGCCTTCCGTGTTCCTA | GATGCCTGCTTCACCACCTTCT |
| *Bact* | GCATGGCCTTCCGTGTTCCTA | ATCGTACTCCTGCTTGCTGA |

**Table S3. Sequence of primers used for RT-PCR and qPCR.**
