# Supplementary material for: Gli3 is a negative regulator of Tas1r3-expressing taste cells
Source: PLoS Genet. 2018 Feb 7;14(2):e1007058. doi: 10.1371/journal.pgen.1007058 (PMC5819828; doi:10.1371/journal.pgen.1007058)
Supplement: S4 Table — (DOCX) [file pgen.1007058.s012.docx]

**Table S4. List of antibodies used.**

| **Antibody** | **Host** | **Cat. no.** | **Research Resource Identifier (RRID)** | **Source** | **Dilution** |
| --- | --- | --- | --- | --- | --- |
| Gli3 (2676A) | Rabbit | － | － | Dr. Suzie Scales, Genetech Inc.,San Fransico, CA | 1:500 |
| TRPM5 | Guinea pig | － | － | Dr. Emily Liman, University of Southern California, Los Angeles, CA | 1:800 |
| PKD2L1 | Rabbit | AB9084 | AB_571091 | Millipore, Billerica, MA | 1:300 |
| T1R3 | Goat | sc-22459 | AB_2255772 | Santa Cruz, Dallas, TX | 1:200 |
| T1R3 | Rabbit | － | － | Dr. Peihua Jiang, Monell Chemical Senses Center, Philadelphia, PA | 1:500 |
| Gustducin | Rabbit | sc-395 | AB_673678 | Santa Cruz, Dallas, TX | 1:500 |
| CAR4 | Goat | AF2414 | AB_2070332 | R&D Systems, Minneapolis, MN | 1:50 |
| NTPDase2 | Rabbit | － | － | Dr. J. Sevigny, Laval University, Quebec, Canada | 1:250 |
| KCNQ1 | Rabbit | sc-22459 | AB_2255772 | Santa Cruz, Dallas, TX | 1:500 |
| KCNQ1 | Goat | SC-10646 | :AB_2131554 | Santa Cruz, Dallas, TX | 1:500 |
| 5-HT | Rabbit | 20080 | AB_572263 | Immunostar, Hudson, WI | 1:1000 |
| Anti-rabbit IgG-Alexa 488 | Donkey | A11008 | － | Invitrogen, Eugene, OR | 1:1000 |
| Anti-rabbit IgG-Alexa 555 | Donkey | A31572 | － | Invitrogen, Eugene, OR | 1:1000 |
| Anti-rabbit IgG-Alexa 647 | Donkey | A31573 | － | Invitrogen, Eugene, OR | 1:1000 |
| Anti-goat IgG-Alexa 488 | Donkey | A21206 | － | Invitrogen, Eugene, OR | 1:1000 |
| Anti-goat IgG-Alexa 555 | Donkey | A21432 | － | Invitrogen, Eugene, OR | 1:1000 |
| Anti-guinea pig IgG-Alexa 647 | Donkey | 706-605-148 | － | Thermo Fisher, Carlsbad, CA | 1:1000 |
